# Bi-Layered, Ultrathin Coating Initiated Relay Response to Impart Superior Fire Resistance for Polymeric and Metallic Substrates

**DOI:** 10.1007/s40820-025-01739-8

**Published:** 2025-04-25

**Authors:** Wei Tang, Qi Chen, Junxiao Li, Xiang Ao, Yunhuan Liu, Lijun Qian, Silvia González Prolongo, Yong Qiu, De-Yi Wang

**Affiliations:** 1 IMDEA Materials Institute, C Eric Kandel 2, 28906 Madrid, Spain; 2 College of Light Industry Science and Engineering, Beijing Technology and Business University, Fucheng Road 11, Beijing, 100048 People’s Republic of China; 3 Materials Science and Engineering Area, Escuela Superior de Ciencias Experimentales y Tecnología, Universidad Rey Juan Carlos, Calle Tulipán s/n, 28933 Móstoles, Madrid, Spain; 4 Escuela Politécnica Superior, Universidad Francisco de Vitoria, Ctra. Pozuelo-Majadahonda Km 1, 800, 28223 Pozuelo de Alarcón, Madrid, Spain; 5 E.T.S. de Ingenieros de Caminos, Universidad Politécnica de Madrid, Calle Profesor Aranguren 3, 28040 Madrid, Spain; 6 Instituto de Investigación de Tecnologías para la Sostenibilidad, Universidad Rey Juan Carlos, 28040 Madrid, Spain

**Keywords:** Flame retardant, Coating, Ultra-thin, Relay response, Battery cases

## Abstract

Developing high-efficient flame-retardant coatings is crucial for fire safety polymer and battery fields. Traditional intumescent coatings and ceramifiable coatings struggle to provide immediate and prolonged protection simultaneously, which limits the applicability. To address this, an innovative bi-layered coating with organic/nano-inorganic additives is inspired by differential response behaviors, enabling relay response effect with both fast-acting and extended protection. Specifically, two layers function continuously in the form of a relay. With a mere 320 microns, the bi-layered coating withstands fire temperatures of up to 1400 °C for at least 900 s. Consequently, the coating effective prevented burn through in aluminum plates and glass fabric-reinforced epoxy resin, which otherwise were burned through in 135 and 173 s, respectively. Meanwhile, the bi-layered coating suppressed the formation and decomposition of solid interface layer in lithium soft-package batteries, leading to prolonged electrochemical stability and fire safety. Additionally, the bi-layered coating with a fast response endows polyurethane foam with rapid self-extinguishing, preventing ignition even under exposure to strong fire of 1400 °C. Shortly, our work offers new insights into the design and development of thin, high-performance, and multi-application flame-retardant coatings.

**Supplementary Information:**

The online version contains supplementary material available at 10.1007/s40820-025-01739-8.

## Highlights

- Relay response of bi-layered coating achieved fast response and extended protection.
- 320-µm coating achieved over 900 s of burn-through resistance.
- 320-µm coating achieved extended electrochemical stability for battery under fire.

**Supplementary Information:**

The online version contains supplementary material available at 10.1007/s40820-025-01739-8.

## Graphical abstract

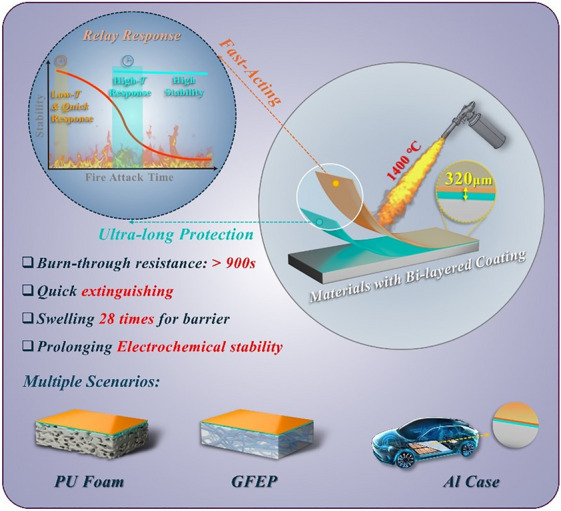

## Introduction

Flame-retardant coatings are effective for enhancing the fire resistance of materials in various industries, including construction, transportation, and electronics [1–4]. Coating flame-retardant technology offers a range of advantages, particularly in its ability to provide surface-level fire protection without significantly altering the underlying properties of the material. By applying a flame-retardant coating, the material is enveloped in a protective layer that acts as a barrier against heat and oxygen, two critical elements needed for combustion [5, 6]. This approach is especially valuable in applications where preserving the original characteristics of the material is required. 

Primarily, coating technology is undoubtedly the optimal solution for metal-based materials, such as aluminum and its alloy [7–11]. In particular, in the field of batteries, aluminum is frequently used for the fabrication of integral pack battery enclosures and laminated pouches for soft-package batteries (SPB). Owing to their relatively low melting temperature around 660 °C and high thermal conductivity of aluminum, it is extremely susceptible to the risk of thermal runaway and explosion of batteries under fire conditions [12–16]. Therefore, the development of high-performance fire-resistant coatings is essential for high safety batteries. Apart from that, for some polymer materials, the advantages of coating technology are also evident. For instance, rigid polyurethane (PU) foam is often used as a thermal insulation material, and the traditional additive flame-retardant method is feasible to raise the flame retardancy efficiency, but significantly weakened the thermal insulation performance, density, and other physical and mechanical properties [17, 18]. Also, applying polymeric coating on the laminate surface has been one of the most popular flame-retardant approaches for fireproofing fiber-reinforced polymer composites [19, 20]. Another example is the fiber-reinforced polymer composites, slowing heat transfer and temperature rise-up through the thickness direction of greater importance than simply lowering fire hazards such as heat and smoke release due to their widely use in structural loading applications [21]. Up to now, several efforts have been made to tackle this challenge with thickness more than one millimeter, which indirectly lowered the strength-to-weight ratio of materials [22–25]. To the best of our knowledge, the research for ultra-thin and highly efficient polymeric coating for metal and polymer materials is still in its infancy with more efforts needing to be made.

Currently, intumescent flame-retardant (IFR) coatings and ceramizable coatings are two typical fire protection solutions, each with distinct mechanisms of action and effectiveness [26–28]. Intumescent system expands at elevated temperatures to form char layers that act as a thermal barrier, protecting the underlying substrate from heat exposure [29–32]. These coatings are composed of three primary components: a carbon source, an acid source, and a gas source [33, 34]. Typically, when the temperature exceeds 300 °C, the acid source decomposes to produce acidic intermediates, which catalyze the dehydration of the carbon source. This process rapidly leads to the formation of a cross-linked char layer, and the gases released by the decomposition of the blowing agent cause further expansion of this layer [35–37]. The expanded char layer provides thermal insulation and reduces heat transfer to the substrate, effectively delaying ignition and slowing flame spread [38–41]. As a result, these coatings exhibit an intumescent flame-retardant effect almost immediately upon exposure to fire. However, a significant drawback is that the char layer, primarily composed of phosphorus, nitrogen, carbon, and oxygen, is not robust enough to withstand prolonged exposure to flames and gradually degrades over time, leading to potential failure. Commonly used intumescent flame-retardant coatings are primarily based on polyphosphate flame-retardant systems, which are characterized by high efficiency, quick response, and low cost [42]. Enhancing the barrier properties and thermal stability of the resulting char layer has been a focal point of ongoing research [43].

Ceramizable flame-retardant coatings, on the other hand, are a novel class of functional materials designed to enhance the fire resistance of materials under high-temperature conditions [44, 45]. These coatings undergo chemical reactions in fire or high-temperature environments to form a dense ceramic layer, providing protection to the substrate. By forming a ceramic layer during a fire, these coatings not only improve the fire resistance of materials but also offer additional structural support, preventing rapid failure under high-temperature conditions [46, 47]. The mechanism of ceramizable flame-retardant coatings primarily relies on phase transformation and chemical reactions at elevated temperatures [48]. The resulting ceramic layer acts as a thermal barrier, effectively impeding heat transfer to the substrate and reducing further combustion and degradation of the material [49]. Silicone-containing matrices are frequently utilized in combination with various fillers and structural modifications to develop ceramifiable materials. For instance, zirconium silicide and montmorillonite enhanced ceramization efficiency, significantly reducing both the linear and mass ablation rates of silicone rubber [50, 51]. Additionally, the introduction of boron oxide and silicon nitride facilitates the transformation of silicone rubber into high-strength, hard ceramics at elevated temperatures [52]. Zirconium-based structures have been employed to modify the molecular architecture of polysiloxanes, enabling the formation of an interpenetrating network with other polysiloxane structures. This modification concurrently enhances both the mechanical properties of silicone rubber and its ceramization capability [53]. Furthermore, the synergistic interaction between silicone-containing foams and multi-scale particles, such as low melting glass powders, promotes the formation of robust porous ceramic structures, which exhibit exceptional long-term thermal insulation when exposed to oxidative environments at approximately 1300 °C [54]. These findings underscore the potential of silicone-containing resins, in combination with inorganic fillers, for the development of high-performance ceramifiable coatings. However, a drawback of ceramizable coatings is that the ceramization process requires a certain period to complete. Consequently, when suddenly exposed to flame attack, an effective ceramic layer may not form in time and even some silicone-based binders are also flammable, causing the coating to burn through and fail.

Further, due to the sensitivity of intumescent flame-retardant systems to inorganic fillers, the option of co-blending intumescent flame retardants with ceramicisable fillers is also not feasible, as high levels of inorganic fillers would destroy the effective intumescent flame-retardant effect [43]. Based on the background, we have considered how to develop coating that can both rapidly respond to achieve flame-retardant effects and withstand prolonged exposure to flames. The inherent advantages and disadvantages of intumescent flame-retardant coatings and ceramifiable coatings inspired us to design a two-layer structure that utilizes a relay response to allow bi-layered coating to function quickly and continuously throughout the duration of a fire attack. In this work, we systematically developed and optimized the formulations of intumescent flame-retardant coatings and ceramizable coatings. More importantly, the different response temperatures, charring speeds, and other characteristics of these two types of coatings inspired us to design a bi-layered structure of coating, which enables our target for developing a coating that can satisfy more application field compared to traditional strategy with a thin level.

## Experiment

### Raw Materials

Ingredients of PU foams (H200-AT, Components A & B) were supplied by Vosschemie Benelux V-Sure, Germany. Ingredients of silicone rubber (ADDV-10, Components A & B, shore A hardness: 10 ± 2, tensile strength: ≥ 2.8 N mm^−2^, elongation at break: ≥ 590%, tear resistance: ≥ 12 N mm^−1^, linear shrinkage: ≤ 0.1%) were obtained from the Faserverbundwerkstoffe Composite Technology, Germany. Piperazine pyrophosphate (PPAP, particle size: 10–30 μm) was provided by Shanghai Research Institute of Chemical Industry Co., Ltd. China. Melamine polyphosphate (BUDIT 610, MPP, Initial decomposition temperature: 340 °C) was purchased from Budenheim Chemicals, Germany. Nano-scaled aluminum oxide (Al_2_O_3_), zirconium oxide (ZrO), zinc oxide (ZnO), titanium dioxide (TiO_2_), and zinc borate (ZB) were bought from Merck KGaA, Germany. Low melting glass powder (D235, GP, pH: 7.9, initial melting temperature: 350 °C) was offered by Anywhere Powder, China. Aluminum phosphate (AlP, 97%, phosphorus pentoxide: 58.0%, aluminum oxide: 41.6%) was obtained from Thermo Scientific, Germany. Talc (NA800, pH: 7–9) was got from the Liaoning Jinghua New Material Inc., China. Biobased epoxy resin (Resoltech 1800 ECO) and cycloaliphatic & aliphatic amine curing agent (Hardener 1804 ECO) were supplied by Resoltech, France. Glass fiber-based woven fabric (UTE 275P, plain weave, 275 g m^−2^) was obtained from Castro Composites, Spain. The preparation process for PU foams, glass fabric-reinforced epoxy resin, and all-solid SPB are detailed in Supporting Information.

### Fabrication of Silicone Rubber Composites

To optimize the preferred formulation for coatings, including ceramic layer and intumescent flame-retardant layer, the silicone rubber materials containing ceramic additives and IFR fillers, respectively, were first constructed and then underwent the fire resistance characterization alone. The formulas of two types of silicone rubber composites are detailed in Tables 1 and 2. The specific preparation process is given in Supporting Information.

**Table 1:** The formula of intumescent fire-retarded silicone rubber composites

Sample	SiR wt%	IFR wt%	Al_2_O_3_ wt%	ZnO wt%	TiO_2_ wt%	ZrO_2_ wt%
SiR	100	/	/	/	/	/
IFR	60	40	/	/	/	/
2Al_2_O_3_/38IFR	60	38	2	/	/	/
2ZnO/38IFR	60	38	/	2	/	/
2TiO_2_/38IFR	60	38	/	/	2	/
2ZrO_2_/38IFR	60	38	/	/	/	2

**Table 2:** The formula of ceramic silicone rubber composites

Sample	SiR wt%	GP wt%	Talc wt%	ZB wt%	AlP wt%
10_G_20_T_5_Z_5_A_	60	10	20	5	5
10_G_5_T_20_Z_5_A_	60	10	5	20	5
10_G_5_T_5_Z_20_A_	60	10	5	5	20
20_G_10_T_5_Z_5_A_	60	20	10	5	5
20_G_5_T_10_Z_5_A_	60	20	5	10	5
20_G_5_T_5_Z_10_A_	60	20	5	5	10
30_G_6_T_2_Z_2_A_	60	30	6	2	2
30_G_2_T_6_Z_2_A_	60	30	2	6	2
30_G_2_T_2_Z_6_A_	60	30	2	2	6

### Application and Thickness of Coating

**Fig. 1:**
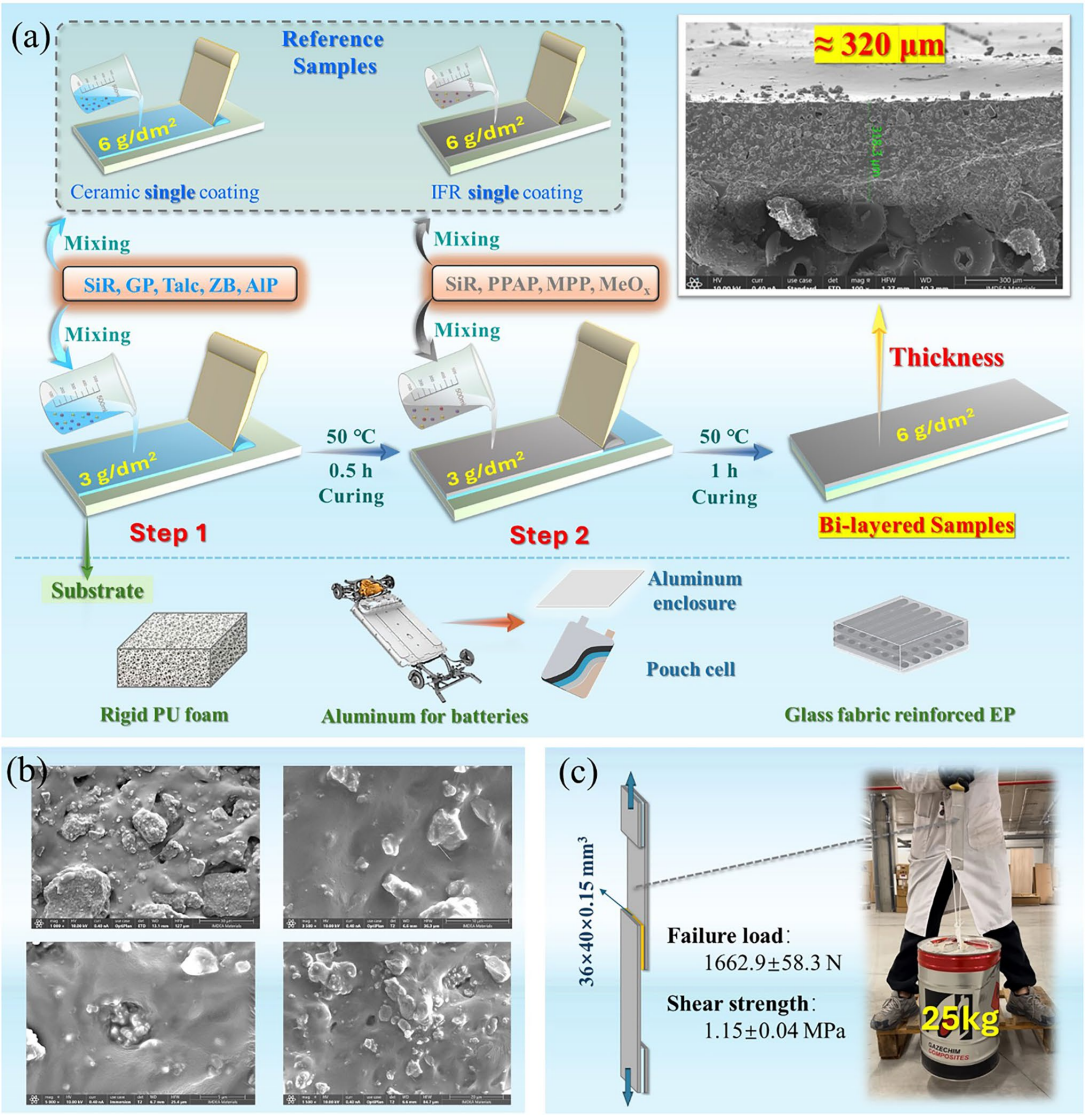
**a** Schematic diagram of bi-layered strategy, **b** micromorphology of fillers in SiR matrix, **c** adhesion property of ceramifiable coating

The optimized silicone rubber formulas were coated on the substrates via the blade method according to the order of applying ceramic layer followed by intumescent layer. The exact steps are shown in Fig. 1a and Supporting Information. The thickness of the bi-layer coating was mere around 320 μm, which reached a quite thin level. The bi-layered structure was proved by EDS mapping in Fig. S3. Meanwhile, the interface morphology between the matrix and the particles demonstrated good compatibility in Fig. 1b. The adhesion performance of inner coating layer was evaluated, and the shear strength was around 1.15 ± 0.04 MPa, as displayed in Figs. 1c, S4 and Video S1.

### Characterization

Detailed characterizations are exhibited in Supporting Information.

## Results and Discussion

### Optimization of Ceramic and IFR Formulations

**Fig. 2:**
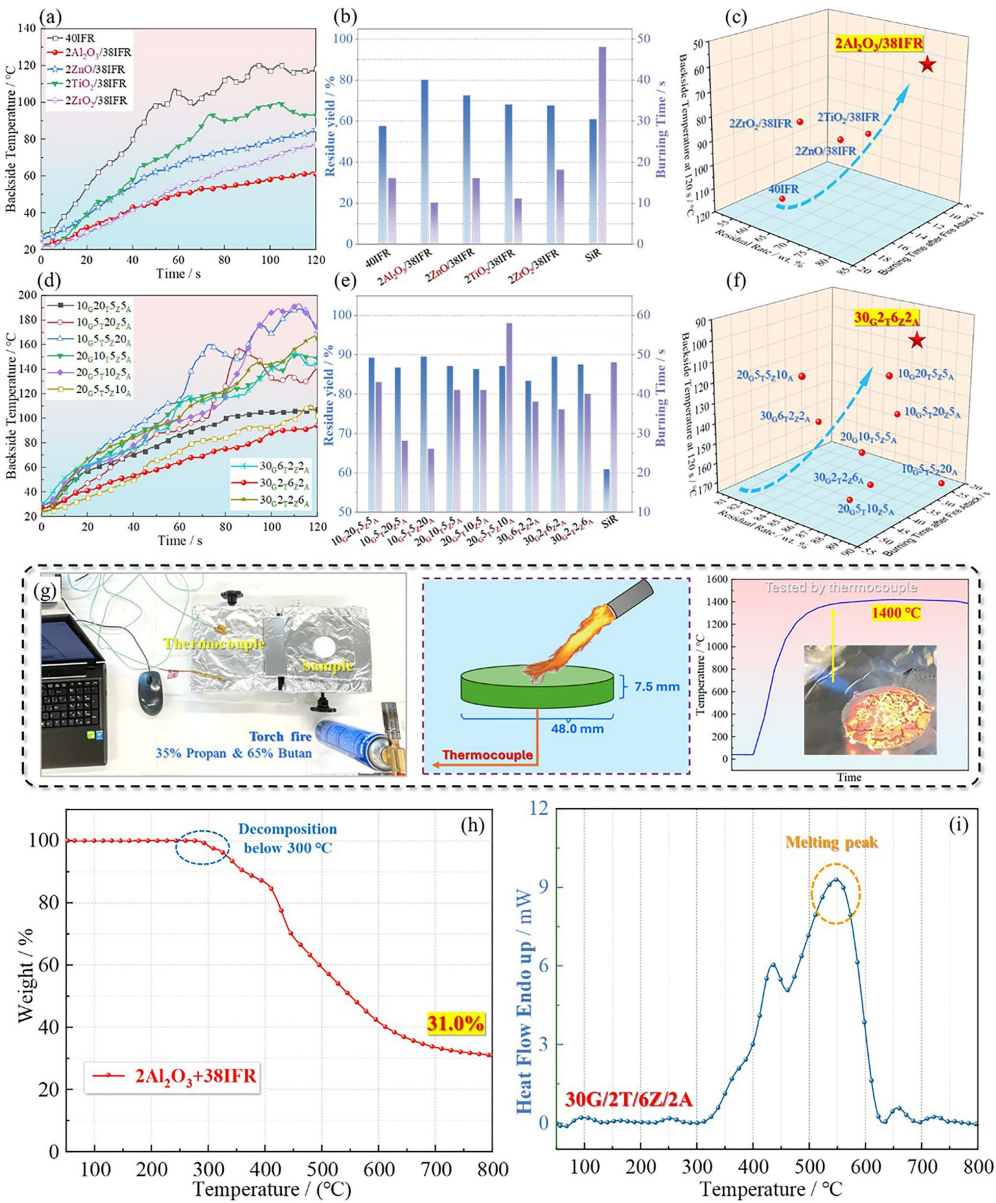
**a**, **d** backside temperature, **b**, **e** residue yield & burning time, **c**, **f** comprehensive fire retardancy of ceramic and IFR silicone rubber materials in **g** assessment method of a torch fire, **h** the TG curve of IFR system, and **i** the heat flow curve of ceramifiable fillers

To optimize the formulas for ceramic layer and intumescent flame-retardant layer, the silicone rubber composites containing these two systems, respectively, were characterized alone via a fire damage test, as displayed in Fig. 2. For the intumescent flame-retardant section, the fire resistance of silicone rubber was improved remarkably by loading piperazine pyrophosphate/melamine polyphosphate system. For instance, the burning time of silicone rubber carried with IFR decreased to 16 s from 48 s of neat silicone rubber. However, the char-forming ability of IFR alone was not high enough, leading to a mere 57.5% residue rate, which was even lower than that of neat silicone rubber. Therefore, synergists are necessary to enhance efficacy. It is obvious in Fig. 2a, b that the 2Al_2_O_3_/38IFR system kept 80% residue yield and realized the self-extinguishing within 10 s. Meanwhile, the backside temperature of sample was only about 60 °C after suffering from the strong torch fire of 1400 °C for 120 s. As exhibited in Fig. 2c, integrating these three parameters, the optimal solution of 2Al_2_O_3_/38IFR can be determined for fabricating intumescent flame-retarded coating layer. For the ceramic silicone rubber materials, the residue yield was significantly improved by loading GP/Talc/ZB/AlP system. In Fig. 2e, neat silicone rubber only performed 60.8% of residue, while other samples loaded with ceramic fillers all exhibited over 80% of residue rate. In particular, the formula 30_G_2_T_6_Z_2_A_ made almost 90% substance remaining in condensed phase. Meanwhile, the residue char of 30_G_2_T_6_Z_2_A_/silicone rubber showed the best barrier effect among these schemes. The backside temperature merely rose to 90 °C in 120-s fire attack, which may be caused by the generation of ceramic structure with excellent thermal insulation. Based on the above concerns, formula 30_G_2_T_6_Z_2_A_ was the best candidate for fabricating the ceramic coating.

According to the results, intumescent flame-retarded silicone rubber responded promptly and resisted flames immediately, while it was time required for the ceramization process of 30_G_2_T_6_Z_2_A_ system. The mechanism was revealed by TGA test, as shown in Fig. 2h, i. The IFR/Al_2_O_3_ decomposed below 300 °C, while the ceramic fillers melted at around 550 °C. As is well known, IFR exerted intumescent flame retardancy through decomposition and crosslinking of ingredients, and ceramic fillers perform a barrier role by melting and adhering to the substrate. Therefore, the designed bi-layered coating from IFR layer and ceramic layer was promising to achieve excellent barrier fire resistance effect by the specificities of fast response at the initial fire stage and high stability at late fire.

### Fire Resistance of PU Foam with Bi-Layered Coating

#### Fire Damage Test with a Fire of 1400 °C

**Fig. 3:**
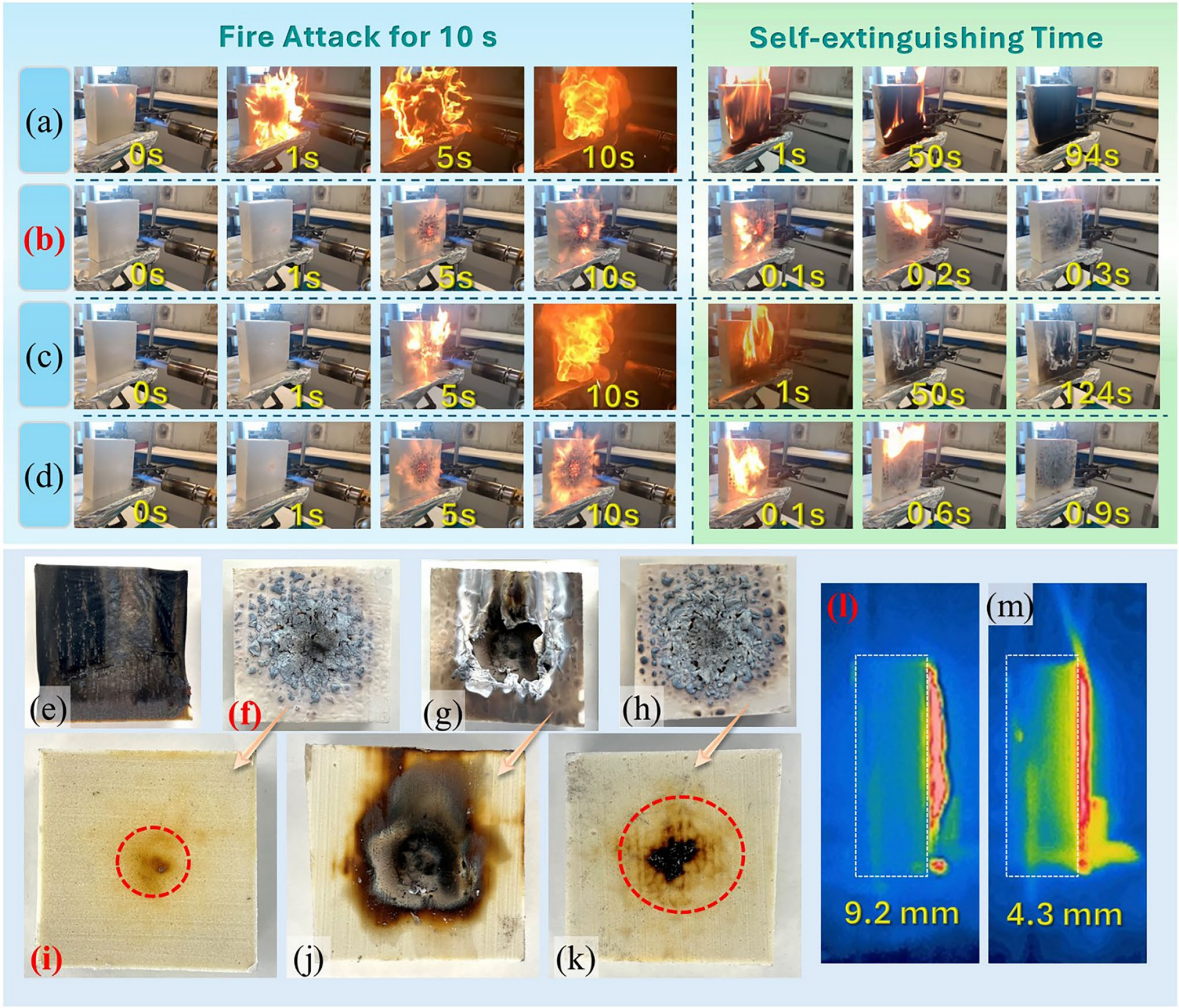
**a**–**d** The test process, **f**–**h** coating & **e, i**–**k** substrate morphology after fire damage, **l, m** infrared thermal images of char layer for neat PU (**a, e**), PU with bi-layered coating (**b, f, i, l**), PU with ceramic single layer (**c, g, j**), and PU with IFR single layer (**d, h, k, m**)

Firstly, the PU foams with and without different coatings were assessed via a strong torch fire, and the test process and relevant results are exhibited in Fig. 3 and Video S2. For neat PU foam, a large flame can be observed when in contact with the fire for only 1 s in Fig. 3a, while other samples with coatings were not ignited and not carbonized immediately, demonstrating that the silicone rubber-based coating materials were more thermal stability than PU foam matrix. During the fire attack of 10 s, the burning intensity of PU without coating increased rapidly, which was caused by the abundant carbohydrate skeletons and multi-porous structure of foam substrate. After removing the torch fire, the self-extinguishing time of neat PU was up to 94 s, resulting in the effects that the surfaces touched by the flames were completely burnt out and carbonized, as shown in Fig. 3e. Conversely, the PU foam coated with a bi-layered coating showed excellent fire resistance. As presented in Fig. 3b, on the one hand, the barrier char layers were generated during the fire devastation process. More importantly, the char layers were thermally stable and kept the integrity, leading to a lower burning intensity, and the PU matrix was completely separated from the flames. On the other hand, the self-extinguishing time for PU with bi-layered coating was mere 0.3 s, almost indicating that the sample cannot be ignited. The main reason was the protective function provided by the char layers in Fig. 3f. Based on these phenomena, it can be speculated that both efficient gas-phase and condensed-phase effects initiated by ceramic/IFR layers achieved superior fire resistance performance. To further assess the thermal insulation of barrier layers formed by different coatings, the coating residues were carefully separated from the PU, and the carbonization degree of matrix is shown in Fig. 3i–k. Surprisingly, the PU matrix protected by the bi-layered coating with hundreds of microns was almost not carbonized, even under the destruction of flames up to 1400 °C, implying that the barrier layer exhibited excellent thermal insulation capacity. Notably, an impressive intumescent ratio occurred from the bi-layered coating in Fig. 3l. Specifically, the maximum height of the swelling char was around 9.2 mm, which was about 28 times the thickness of the bi-layered coating. Further, the expansion ratio of char layers by bi-layered coating was remarkably higher than that of char layers by IFR single layer, even though the percentage of IFR additives in bi-layered coating was 50% lower than that in IFR single layer. The possible reason was that the silicone rubber portion of the internal ceramifiable layer is susceptible to more rapid decomposition when attacked at elevated temperatures, as the ceramifiable filler hardly promotes silicone rubber charring, unlike IFR system. It can be inspirated by the following thermal analysis. Based on these reasons, the PU protected with the bi-layered coating possessed outstanding fire safety properties.

For reference samples of PU coated with single layer, the fire resistance and efficacy of barrier effect were varying. As shown in Fig. 3c, the ceramifiable layer cannot endow PU foam with good fire resistance alone. When the ceramifiable coating suffered from the fire attack, the residue layer was generated first and then burned through. In this case, part of PU matrix was directly exposed to the torch fire, thus resulting in a strong burning intensity, such as the blaze at 10 s of fire application. After the fire attack, the matrix was apparently ignited and kept burning for 124 s, which was even longer than the self-extinguishing time of neat PU. The continuous release of decomposition products of PU components from the cracks in the generated ceramic layer is responsible for this long self-extinguishing time. Moreover, the barrier layer was destroyed seriously during the fire attack and burning process, as displayed in Fig. 3g. Owing to the defects in the residue, severe carbonization phenomenon occurred with the PU matrix in Fig. 3j. From the thermal response aspect, the melting point of the ceramic filler was high to around 550 °C in Fig. 2i, which implied that the ceramic melt layer would not be shaped immediately when attached to fire. Consequently, according to these behaviors, a single ceramic coating cannot develop a ceramic layer quickly within an instant, making it susceptible to damage by strong fires, thereby failing to provide effective protection to the PU substrate. As to the efficiency of PU with single IFR coating in Fig. 3d, the stable char layers were built during the fire attack process, which exerted a good barrier effect and led to a mere 0.9 s of self-extinguishing time. The main reason was that the decomposition of IFR/Al_2_O_3_ system was below 300 °C, which facilitated a rapid response to fire. Specifically, the interaction among the different components in flame-retardant system promoted the formation of cross-linked char layers to fulfill the barrier effect, as shown in Fig. 3h [43]. Meanwhile, a specific gas-phase effect may also contribute to the short self-extinguishing time. Nevertheless, the morphology in Fig. 3k exhibited a more severe carbonization degree, especially compared to the PU matrix protected by the bi-layered coating. These phenomena demonstrated that the single IFR layer was able to achieve a quick flame retardancy to PU, but the capacity of thermal insulation for formed char layers was not high enough to perform good thermal insulation. Contrastingly, the bi-layered coating gathered both advantages of rapid response from IFR layer and high thermal stability/thermal insulation from ceramic layer, resulting in effective and long-lasting protection effect.

#### Fire Damage Test with a Fire of 1000 °C

To thoroughly evaluate the efficacy and benefit of the bi-layered coating, the built samples were characterized under another different fire condition with the temperature of around 1000 °C. The test process and burning situation are detailed in Fig. S9 and Video S3. The bi-layered coating enabled PU excellent flame retardancy and no fire spread, as shown in Fig. S9b. In particular, even though the fire application time was up to 60 s, the self-extinguishing time decreased to mere 10 s from the 237 s of neat PU, and the formed barrier layer retained its shape stability and denseness during the whole process, which all implied that the matrix under the coating was not ignited. For the IFR single layer, the problem was that the generated char layers were not thermally stable enough, especially under the long-term fire attack. Hence, the matrix covered by the IFR coating was seriously carbonized during the test. On top of these, a conclusion can be given that the bi-layered strategy was able to achieve the outstanding barrier effect and flame retardancy with hundreds of microns.

#### Comprehensive Fire Safety Performance in Cone Calorimeter Test

**Fig. 4:**
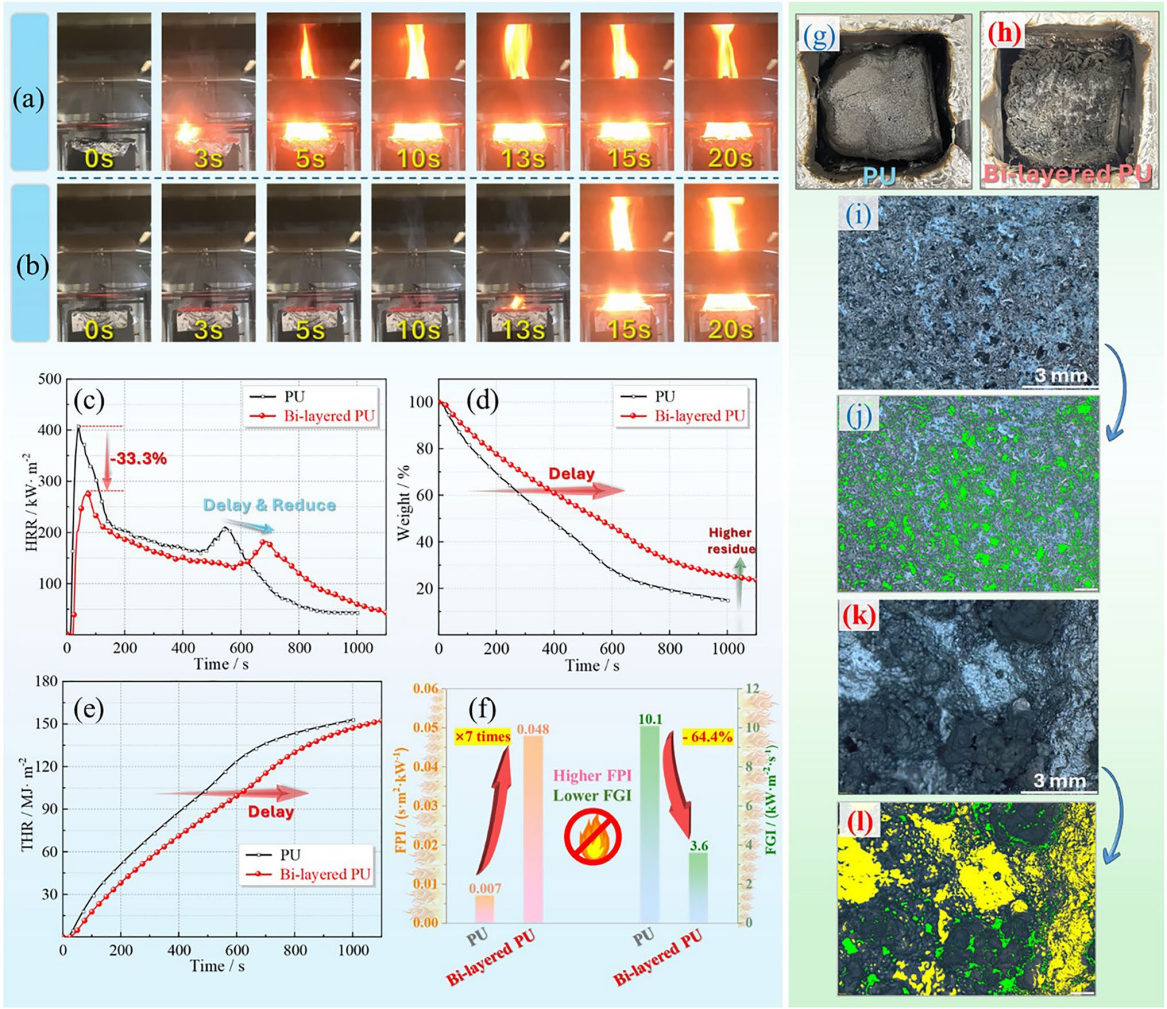
Initial test condition of **a** PU and **b** PU with bi-layered coating, **c** HHR, **d** weight loss, **e** THR curves, **f** fire safety index, and the surficial residue morphology of **g, i, j** PU and **h, k, l** PU with bi-layered coating

To quantitatively estimate the fire safety performance of PU with bi-layered coating, the cone calorimeter facility was used to provide some crucial parameters and charts, as shown in Table 3 and Fig. 4. First of all, as displayed in Fig. 4a, b a significant improvement was that the PU with bi-layered coating exhibited 13 s of time to ignition (TTI), while PU was ignited within 3 s, proving that the coating delayed the burning generation or made it more difficult to trigger fire. And this enhancement was caused by a coating with mere hundreds of microns, which fully demonstrated the rapid charring behavior and outstanding barrier effect from the coating with bi-layered structure. Once ignited, the intensity of the fire escalated rapidly owing to the continuous heat irradiation of 50 kW m^−2^. Due to the barrier effect from the formed char layers, the peak heat release rate (PHRR) of bi-layered PU sample was around 33.3% lower than that of neat PU, as shown in Fig. 4c. More importantly, the time to PHHR (TTP) was also significantly increased. Specifically, bi-layered PU material achieved 75 s of TTP, which was almost two times that of neat PU. Further, bi-layered PU kept lower combustion intensity before 600 s compared to neat PU, implying that the formed char layers provided an effective and durable protection. The coating also delayed and reduced the secondary peak of HRR curve at about 700 s, illustrating a much lower burning speed was undergoing. Moreover, based on the data of PHRR, TTI, and TTP values, two indexes can be calculated to reflect the difficulty of ignition, intensity of combustion, and speed of fire spread in a comprehensive manner, including fire performance index (FPI = TTI/PHHR) and fire growth index (FGI = PHHR/TTP). Higher FPI and lower FGI signified superior fire safety performance. As exhibited in Fig. 4f, bi-layered PU displayed a 0.048 s m^2^ kW^−1^ of FPI and 3.6 kW m^2^ s^−1^ of FGI, which were around 6 times higher and 64.4% lower than that of PU, respectively. Besides, Fig. S11 and Table S2 indicate that bi-layered coating was more efficient in inhibiting combustion, delaying weight loss and heat release. All these results indicated that the application of bi-layered coating enabled an inhibition in fire occurrence and propagation notably.

**Table 3:** The typical parameters from cone calorimeter tests

Sample	TTI /s	TTP /s	PHRR /kW m^−2^	THR /MJ m^−2^	TSR /m^2^ m^−2^	R_end_ /%	PMLR /g s^−1^	Av-EHC /MJ kg^−1^
PU	3 ± 0	40 ± 0	405 ± 4	151.4 ± 1.6	3770 ± 104	16.0 ± 1.0	0.202 ± 0.010	23.4 ± 0.1
Bi-layered PU	**13 ± 0**	**75 ± 5**	**270 ± 10**	151.5 ± 0.8	4010 ± 120	**24.5 ± 1.1**	**0.130 ± 0.008**	23.2 ± 0.2

Bolded numbers represent significantly improved fire safety performance parameters

The weight loss curve in Fig. 4d revealed the charring behavior and ability of materials during the whole combustion process. It was obvious that the bi-layered coating suppressed the weight loss tendency, which may be contributed by the thermal insulation of the formed char layers, thus leading to a slow decomposition speed of matrix. As also detailed in Table 3, the peak of mass loss rate (PMLR) of bi-layered PU was around 0.130 g s^−1^, which was 35.6% lower than that of neat PU, also proving the lower burning speed. Therefore, the heat release was also delayed by the coating, as indicated in Fig. 4e. Moreover, the residue rate (*R*_end_) increased from around 16.0% of PU to 24.5% of bi-layered PU. The possible reasons for the around 0.5 times growth were as follows. On the one hand, the charring ability of ceramic and IFR fillers was much higher than PU matrix, especially the residue rate of ceramic fillers was over 97%, as exhibited in Fig. 7b. On the other hand, the formed barrier layers were able to block the heat and gas transfer, thus resulting in lower degrees of thermal oxidative degradation for the matrix protected by the bi-layered coating. As to the total heat release (THR) and total smoke release (TSR), there were no remarkable distinguishes between PU and the coated PU sample, because the thin coating exerted the fire resistance effect individually. The main function of the coating was delaying and inhibiting the decomposition and burning behavior. To clarify the reason for the excellent barrier effect of the bi-layered coating, the surface morphology of residual char was observed, and the holes were labeled green. As presented in Fig. 4g, f, the surface char layer of the PU exhibited multiple cracks, whereas the matrix of the bi-layered PU was covered by the char layers from the degradation of the ceramic/IFR coating. As a result of the gasification and combustion for PU, the loose and porous char was generated, which is illustrated in Fig. 4i, j. On the contrary, the quantity of holes on the surface of coating char layer was much less owing to its excellent stability, as exhibited in Fig. 4k, l. Furthermore, the surficial char in Figs. 4l and S12 presented a kind of secondary structure, which was composed of char from IFR layer (black part) and ceramic char from ceramic layer (yellow part). This secondary structure ensures a better blocking effect when exposed to high temperatures for a long time. In short, the bi-layered structure of coating was able to exert quick charring behavior at the initial stage and keep the integrity of the barrier layers, thus endowed the PU foam outstanding fire safety performance.

### Fire Resistance of Aluminum Enclosure and SPB

#### Burn-Through Resistance of Aluminum Plate

**Fig. 5:**
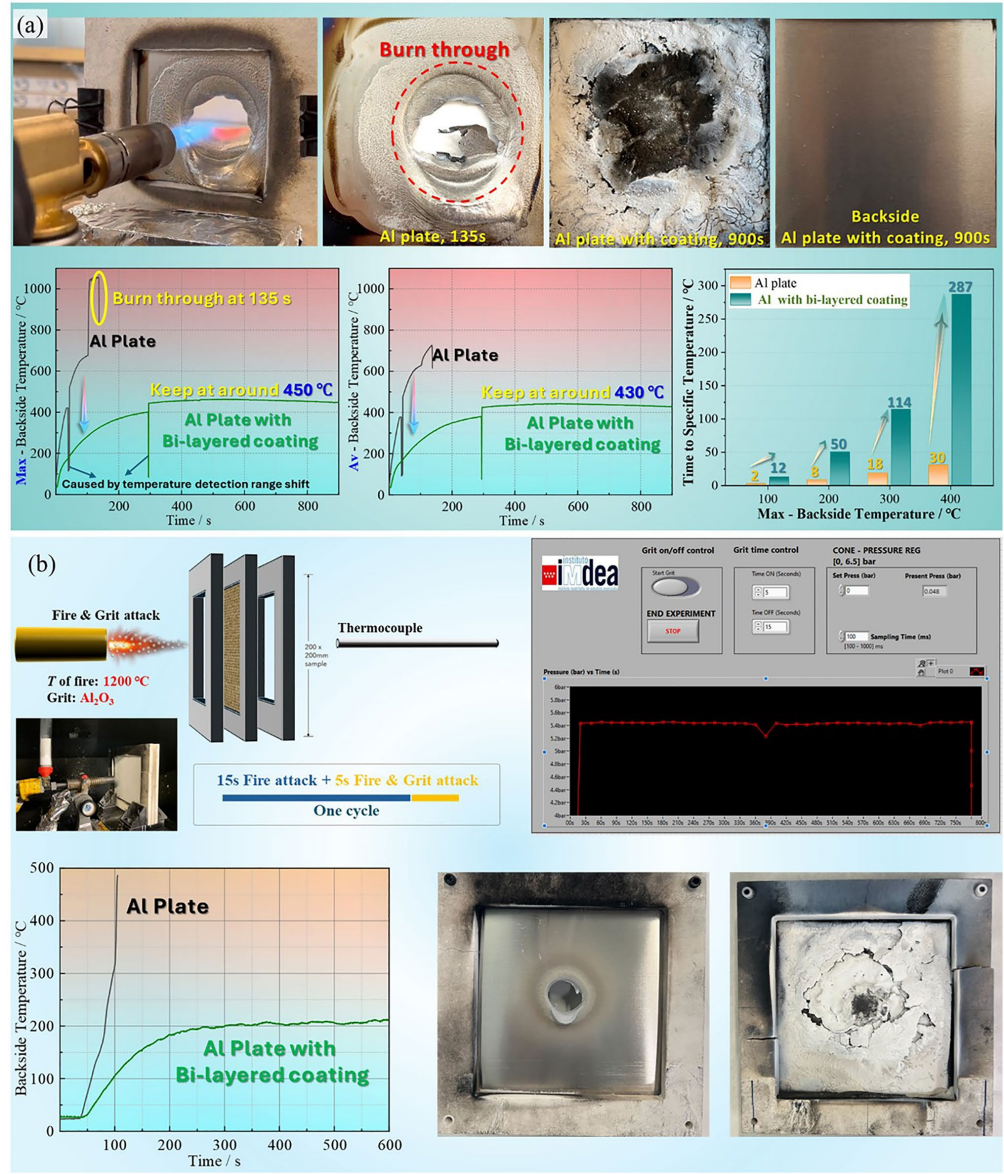
Evaluation method and results of **a** torch fire test and **b** UL 2596 test

To widen the potential application of the constructed bi-layered coating, the bi-layered coating with mere hundreds of microns was applied on the aluminum plate and characterized by the burn-through test. The test process and results are shown in Video S4 and Fig. 5a. It was obvious that the Al plate without coating can be easily burned through in 135 s, which was caused by low melting point of aluminum under the continuous fire impingement. More specifically, the backside temperature of Al plate increased suddenly from around 100 s, indicating the beginning of melting phenomenon. Therefore, outstanding thermal insulation effect of the protective layer was essentially required for avoiding the melting and burn-through outcomes. Meanwhile, the durability of the generated layers was also crucial to guarantee the integrity of substrate, especially under a strong fire with over 120 °C. Surprisingly, the test findings illustrated that the thin bi-layered coating achieved excellent protection level to the matrix. Meanwhile, the aluminum plate with fire resistance coating was not ruined by the fire attack in 900 s, which was completely contributed by the quite stable char layers from the silicone rubber-based coating. More detailly, Fig. 5a presents that the backside temperature of Al plate with coating increased much more slowly compared to the reference sample, and finally, the maximum and average temperature of backside reached up to 450 and 430 °C, respectively, without further rising anymore until test finished. The surficial char layer kept the integrity and compactness during the whole fire destruction, especially at the later stages of test, which may be caused by the extreme stability of the ceramic char layer. All above data fully proved that the barrier layer formed by the bi-layered coating was highly thermal stability and thermal insulation, thus realized an excellent charring protection efficiency to Al plate. Besides, the bi-layered flame-retardant coating delayed the time to specific temperatures of the sample backside. For instance, two hundred degrees is a relatively critical temperature, above which the risk of battery explosion rises dramatically. In this case, it only took 8 s to reach 200 °C of max temperature for the reference sample without coating, while the bi-layered coating enabled this time increase by 5 times. This improvement signified more time would be earned for escaping under some extremely risky conditions. In addition, UL 2596 is used to evaluate the structural stability of coatings under fire conditions as the test includes sand impact, as shown in Fig. 5b. The pure Al plate was broken through in the 5th cycle test, while the Al plate with bi-layered coating withstands more than 30 cycles attack. And the char residue exhibited relatively integrated morphology. It was worthy to mention that the temperature of backside was kept around 200 °C at later stage, even though the temperature of fire is high to 1200 °C.

#### Electrochemical Stability of SPB with Bi-Layered Coating

**Fig. 6:**
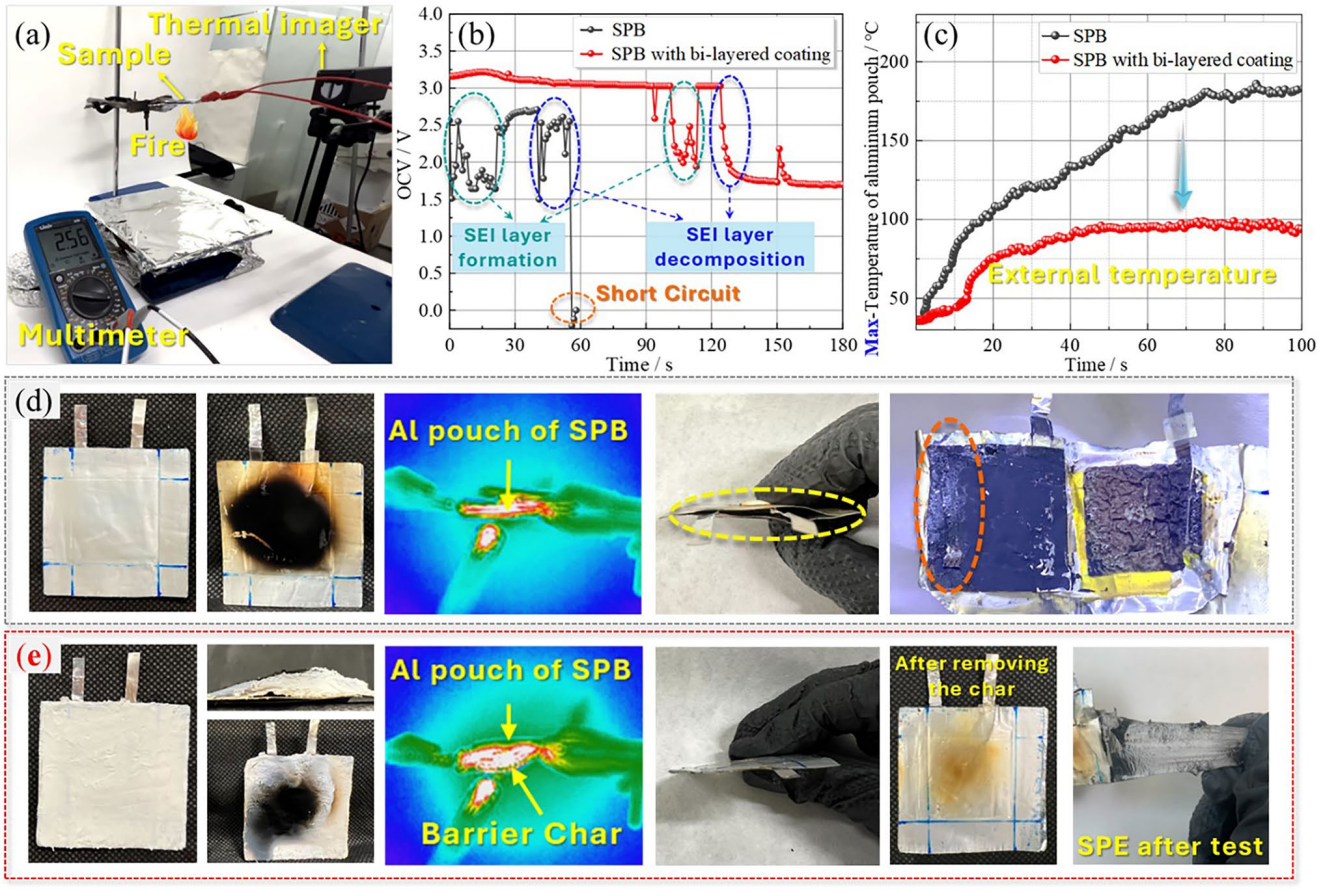
**a** Test method for electrochemical stability under fire condition, **b** OCV curves during the fire, **c** the maximum temperature of pouch side surface, and the relevant digital and thermal images for **d** SPB and **e** SPB with bi-layered coating

To evaluate the protection behavior of the bi-layered coatings for the all-solid-state soft-package batteries, the burning tests were operated by the setup in Fig. 6a under the monitoring of a thermal infrared camera, while the open-circuit voltage (OCV) was measured by a multimeter, which was an important indicator to check the short circuit for batteries. According to the Nernst equation, the OCV value changed with the concentration of the components inside the batteries [54, 55]. Hence, to guarantee the OCV variation only from the concentration, the SPB used for combustion testing was fresh, without charging–discharging operation. According to the *Nernst* formula, the OCV was affected by the concentration of the lithium ions surrounding the electrode materials. So, the different OCV of the two cells without any charge or discharge was possibly related to the different lithium ions concentration at electrodes. The initial OCV for the two SPBs was 2.56 and 3.17 V before burning tests, respectively. Once the SPBs were heated by the flame, the temperature surrounding the cross section of the two batteries increased continuously at different rates, as shown in Fig. 6c. Apparently, the temperature of the SPBs with bi-layered coating increased at a much slower rate and remained stable, while the uncoated SPB rose throughout the process. Meanwhile, the OCV values during the burning process also presented different trends, as shown in Video S5. The coated SPB delivered a more stable voltage of up to ~ 105 s, whereas the voltage of the uncoated SPB jumped significantly from the beginning to ~ 25 s. This distinct tendency of OCV values should be related to the internal reaction of the batteries. The possible reason for jumping was that the internal temperature increased over time, and lithium concentration changed significantly due to the accelerated lithium-ion transport in the solid polymer electrolytes (SPEs) [56]. When the internal temperature reached the melting point of PEO-based SPEs (~ 60 °C), the SPEs with a good fluidity inevitably react with the Li anode more sufficiently to generate the solid interface (SEI) layer, which can effectively protect the Li anode [57]. This resulted in the first fluctuation of the OCV at 0 s for uncoated SPB and 105 s for protected SPB, respectively, illustrating that the SPE in uncoated SPB started melting at the beginning of burning, while the SPE started melting after 105 s in coated SPB. This hysteresis of the time for SPE melting resulted from the protection of the bi-layer coating. Then, the OCV of two SPBs was restabilized after SEI formation at 25 and 105 s, respectively. However, the components of the SEI layer generally included some low molecular weight of polyester or polyether; their thermal stability was not good enough to exist at a higher temperature (> 90 °C) [58]. The SEI layer would start decomposing as the temperature rose (~ 38 s of the black curve), causing the second-time unstable data in the OCV curve, ~ 38 s for the uncoated SPE, and ~ 126 s for coated SPE. Finally, the short circuit happened in uncoated SPB at around 58 s. Compared to uncoated SPB, the data stabilized another two times for coated SPB after the drop at 126 s, strongly demonstrating that bi-layer coating extended the time of SEI layer decomposition due to its thermal insulation effect.

After the burning test, the uncoated SPB bulged, and the coated one was intact (Fig. 6d, e). This may be caused by the decomposed gas (e.g., CO_2_) of the components inside the batteries, such as the SEI layer [16]. On the contrary, due to the protection of the bi-layered coating, the decomposition of the SEI layer was insufficient for coated SPB, enabling the battery to remain intact. To further confirm the protective effect of bi-layered coating on the SPB, the two SPBs were disassembled in a glove box within 2 h. From the last picture of Fig. 6d, e, the lithium metal and SPE had already been melted in the uncoated SPB pouch cell, indicating the internal temperature of the uncoated SPE at least reached the melting point of lithium metal that is 180.5 ℃. However, the SPE still maintained its mechanical strength and flexibility in the coated SPB, as displayed in Video S6. It was ascribed to the effective protection of the bi-layered coating, indirectly proving that the internal temperature of the coated SPE had not reached the melting point, which would be lower than 60 °C. From this aspect, the bi-layered coating induced a huge temperature difference of 120.5 °C at least. These results demonstrated that the bi-layered coating had an effective protective effect on the SPBs, showing its promising potential for the development of fire safety energy storage devices.

### Flame Retardancy of GFEP with Bi-Layered Coating

#### Burn-Through Resistance

The coating strategy is also a preferred flame-retardant solution which deteriorated little on the mechanical properties of the polymer composites. Therefore, the bi-layered coating was applied on the surface of GFEP, which was widely used in lightweight structural loading applications. The burn-through test process and main results are displayed in Video S7 and Fig. S14. The bi-layered coating significantly delayed the decomposition of EP matrix and avoided the burn-through phenomenon of glass fabric. As shown in Fig. S14b, there was no obvious combustion behavior in the initial stage with the sample, which was mainly contributed by the excellent nonflammability of the silicone-based coating. With the continuous heat transfer through the thickness, the EP matrix started decomposition, and the generated flammable volatile transmitted through the barrier layer and burned simultaneously. Nevertheless, the surficial char layers were quite stable and retained integrity upon the impulsive torch fire damage, thus slowing down the decomposition of EP matrix and preventing the direct destruction to glass fabric from the torch fire. As displayed in Fig. S14c, d, the maximum and average backside temperature maintained at around 500 and 400 °C from 200 to 900 s, respectively. From Fig. S14, the carbonization degree of GFEP backside was significantly suppressed by the bi-layered coating. Furthermore, Fig. S14e illustrates that the barrier layer formed by the bi-layered coating remarkably increased the time requirement to specific temperature, suggesting that the structural integrity of the reinforced EP composites will be kept for a longer time under the fire conditions.

#### Comprehensive Fire Safety Performance in Cone Calorimeter Test

The main results of cone calorimeter test are detailed in Table S3 and Fig. S16. Firstly, it was quite remarkable that the application of bi-layered coating decreased the burning intensity of the sample, especially at the initial stage of the combustion. By comparing Fig. S16a, b, the flame of bi-layered GFEP started to be weaker from 15 s compared to the flame of GFEP, which signified that the char layer was formed quickly to exert a barrier effect. With the gradual generation of the compact char layers, the combustion intensity was further reduced, which was also reflected from HRR curves of Fig. S16c. The first peak value of HRR for bi-layered GFEP was only 168 kW m^−2^, which was 44.2% lower than that of neat GFEP. Notably, TTP was also delayed from 105 s of GFEP to 125 s of sample with bi-layered coating. Besides, the PHRR was also slightly decreased by the bi-layered coating. All these behaviors were quite benefit for controlling the propagation of fire and saving more time for escaping. The primary cause was that the application of coating influenced mass loss and burning behaviors in both gas-phase and condensed-phase parts. On the one hand, the generated char layers dramatically delayed the thermal decomposition of EP matrix, which was found from the mass loss curves in Fig. S16d. In addition, the maximum decomposition speed was also suppressed by the barrier layer. In other words, GFEP with the bi-layered coating can decompose later and slower when damaged by a heat source. Meanwhile, owing to the phosphorus-based IFR system, some P-containing free radicals were released from the polyphosphate skeleton to exert a quenching effect and lead to insufficient combustion reaction. Hence, the av-EHC of material was also reduced. As to the higher THR and TSR values, the reason might be related to the thermal decomposition and burning behavior of bi-layered coating material in bi-layered GFEP samples.

### Thermal Decomposition Behavior

**Fig. 7:**
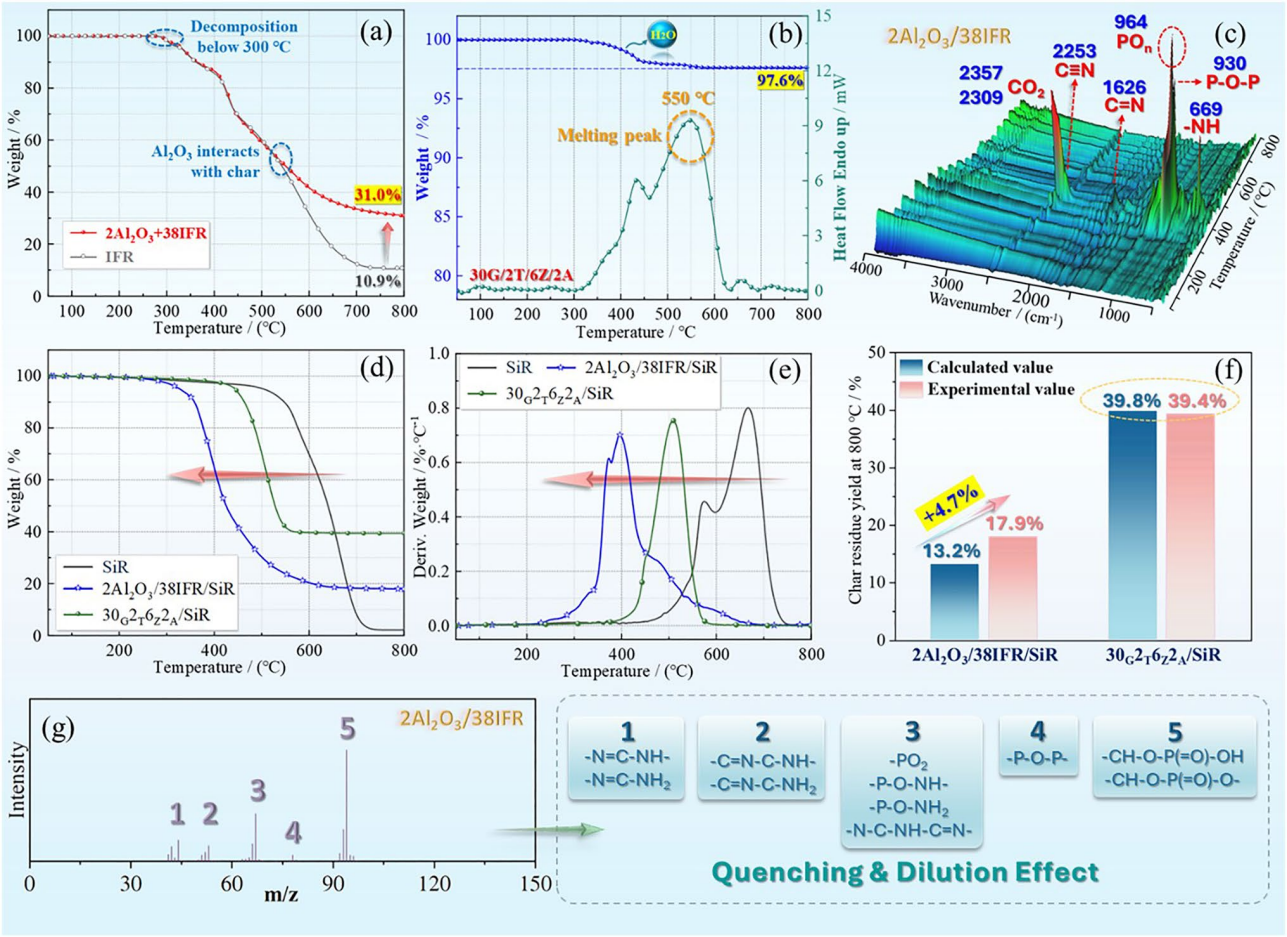
**a** TG curves of IFR and 2Al_2_O_3_/38IFR fillers, **b** TG and heat flow curves of ceramic filler, **c** real-time FITR spectra of 2Al_2_O_3_/38IFR decomposition products, **d** TG and **e** DTG curves of silicone rubber composites, **f** the residue yield at 800 °C, and **g** the main result of py-GC/MC test for 2Al_2_O_3_/38IFR system

In order to thoroughly investigate the mechanism of the bi-layer coating for efficient fire protection, the TGA test was conducted first. For the surficial intumescent flame-retarded layer, the degradation of flame-retardant system started from below 300 °C in Fig. 7a, while the test temperature of torch fire was high to 1400 °C. Hence, the lower decomposition temperature of flame retardant made it possible for responding to attack fire quite timely. Furthermore, the optimized alumina synergist significantly improved the charring ability of IFR. Specifically, the residue yield of 2Al_2_O_3_/38IFR was around 31% at 800 °C, which was three times compared to that of IFR alone. The marked improvement was caused by the higher stability of char residue at high-temperature level. More detailed information was found in the TG curves. A comparison of the weightless behavior showed that the two curves almost coincide at below 550 °C, whereas once the temperature reaches above 550 °C, the two curves trend in separate directions. Therefore, it can be deduced that the alumina compound interacted with the char from IFR at around 550 °C, thus leading to the higher heat resistance of crosslinked char residue. For the internal ceramic layer, the response temperature (melting point) was around 550 °C, which was apparently higher than that of IFR system. Meanwhile, it was time required for the ceramization process. Hence, it was reasonable to design the bi-layer structure with outer IFR layer and inner ceramic layer. The ceramic transformation was undergoing when the IFR system rapidly turns into intumescent char layers to act as a barrier against flame attacks. Additionally, a surprising parameter was the residue rate of ceramic fillers, which reached 97.6% at 800 °C, indicating the ceramic layer was extremely stable. Accordingly, the ceramic layer was able to keep integrity as a second barrier when the IFR layer degrades with the continued flame attack. Based on the above analysis, it can be concluded that the advantages of these two sorts of thermal degradation behavior are combined in the design of the bi-layer coating structure, resulting in excellent flame retardancy and long-term fire resistance performance.

In addition, Fig. 7c, d displays the TG and DTG curves of silicone rubber materials with intumescent flame retardant and with ceramic fillers. Firstly, the charring yields of silicone rubber materials were apparently increased by the modification of IFR and ceramic fillers. The neat silicone rubber showed mere around 2% of residue rate at 800 °C, while 2Al_2_O_3_/38IFR and 30_G_2_T_6_Z_2_A_ imparted the silicone rubber to 17.9% and 39.4%, respectively. Meanwhile, some calculation related to the residue yield of composites was conducted to speculate the interaction of flame-retardant fillers and silicone rubber matrix based on the residue yields of flame retardant themselves and silicone rubber itself, and the main results are demonstrated in Fig. 7e. In calculation, the residue rate of 2Al_2_O_3_/38IFR/SiR and 30_G_2_T_6_Z_2_A_/SiR would be 13.2% and 39.8%, respectively, if there was not any interaction between introduced fillers and silicone rubber substrate. As a matter of fact, 2Al_2_O_3_/38IFR/SiR performed a 17.9% residue yield, which proved that the intumescent flame retardant degraded and reacted with the skeleton of silicone rubber matrix during the burning decomposition process. As to the 30_G_2_T_6_Z_2_A_/SiR sample, the calculated and practical values were almost identical, signifying that the chemical structure of silicone rubber may not participate in the ceramization reaction of fillers. The 30_G_2_T_6_Z_2_A_ mixture and silicone rubber each followed their own decomposition behavior. Besides, the DTG curves exhibited the temperature of maximum decomposition speed (T_d-max_) of materials, which was employed to evaluate the catalytic decomposition behavior of additives. The T_d-max_ of pure silicone resin was around 670 °C. With the loading of additives, the T_d-max_ values of both 2Al_2_O_3_/38IFR/SiR and 30_G_2_T_6_Z_2_A_/SiR were evidently reduced. Particularly, the 2Al_2_O_3_/38IFR/SiR only performed around 400 °C of T_d-max_. Combined the residue yield and decomposition temperature, it concluded that 2Al_2_O_3_/38IFR system initiated the thermal decomposition of silicone rubber matrix in advance and then chemically bonded with the silicone-based skeleton to generate crosslinked char. As for the 30_G_2_T_6_Z_2_A_/SiR sample, the ceramic mixture catalyzed the premature decomposition of the matrix but might not affect the degradation path of silicone rubber.

### Gas-Phase Flame-Retardant Effect

For the inner ceramic layer, the ceramic additives almost did not decompose under high temperature. As a result, there was no gas-phase effect to create. As the external layer, the intumescent flame-retarded silicone rubber not only played a barrier role, but also exhibited excellent gas-phase flame-retardant effect, which was also found from the flash-out phenomenon in the torch fire test for intumescent flame-retarded silicone rubber and bi-layered PU foam. The self-extinguishing behavior caused by the intumescent flame-retardant system undoubtedly contributed to the fire safety performance of the coating, especially for the nonflammability. Figure 7c displays the real-time FTIR spectra of released fragments from TGA instruments. As listed in the chart, the thermal decomposition products of 2Al_2_O_3_/38IFR systems were mainly composed of phosphorus-based and nitrogen-based structures, such as PO, P-O-P, C=N, C≡N, and -NH [59–61]. Furthermore, py-GC/MS characterization provided more detailed information about the chemical structure, as shown in Fig. 7g. The main fragments included phosphorus-containing structures, nitrogen-containing structures, and phosphorus/nitrogen-containing structures. Thereinto, phosphorus-containing radical (PO_2_) enabled the quenching effect to block the burning reaction [62, 63]. Other fragments were able to exert the dilution effect to lower the concentration of flammable substances. In addition, some phosphorus/nitrogen-containing structures containing P-O-N and P-O-C proved that full interaction occurred among the different flame-retardant groups—phosphorus acid, piperazine, and melamine, which corresponded to acid, carbon, and gas sources [64, 65]. Therefore, the quenching and dilution working simultaneously made it possible to inhibit the spread and development of fire, especially in the initial stages of exposure to fire.

### Charring Behavior of IFR and Ceramic Systems

**Fig. 8:**
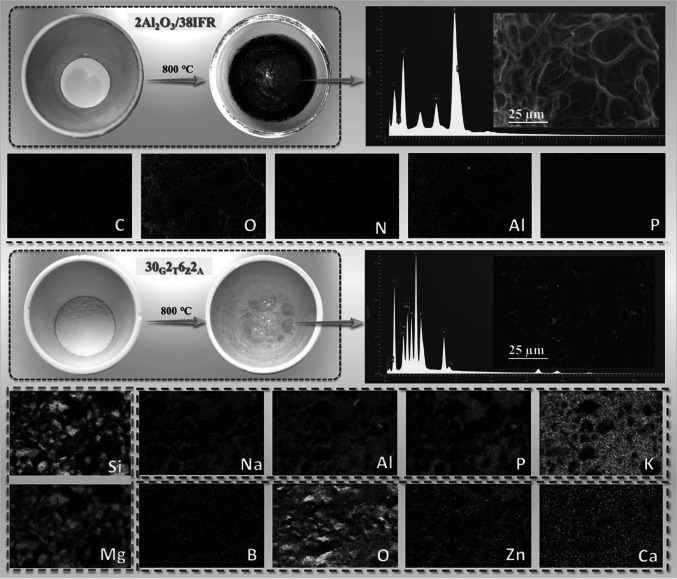
Status of intumescent flame-retardant and ceramic fillers before and after muffle calcination, the surface micromorphology, and EDS elemental distribution of the remaining residues

For the bi-layered coating, the expandable performance and ceramization transformation were quite essential for efficiency. To assess the relevant behaviors, the intumescent flame-retardant mixture and ceramic fillers were conducted by the muffle calcination with the temperature of 800 °C, respectively. As shown in Fig. 8, the 2Al_2_O_3_/38IFR powders swelled more than tenfold in volume after the heat treatment, indicating its excellent intumescent capacity. The surprising swelling ratio of IFR system was the main reason for the superior intumescent flame-retardant effect of the bi-layered coating in Fig. 3l, especially at the initial stage of the fire attack. Theoretically, the higher expansion ratio was beneficial for forming multi-layered char with more closed-cell structure, which was composed of char skeleton and air composition. It is well known that air is a medium with low thermal conductivity. Accordingly, this type of char structure with a high expansion multiplier facilitates excellent thermal insulation in the pre-fire period. Also, the char produced by intumescent flame-retardant system is characterized by a dense and flexible structure, which enables it to perform excellent barrier functions for blocking substance transmission. Moreover, the EDS mapping provided the elemental distribution conditions. Specifically, carbon, nitrogen, oxygen, aluminum, and phosphorus elements were distributed homogeneously in the char structure, indicating the full interaction among different components during the burning process. In particular, the alumina particle was not found in the SEM image of surficial char, but even distributed in the surficial char as the aluminum element, which proved alumina elevated the intumescent flame retardancy of IFR by chemically bonding with P/O/N/C-based skeleton. This speculation was consistent with the evidence in Fig. 7a.

For the ceramic fillers, the formula mixture turned its status from the powder to the glassy after high-temperature exposure, which demonstrated that the ceramization reaction occurred among different raw ingredients. Further, the generated ceramic layer exhibited good continuity and integrity at both the macroscopic and microscopic levels. Owing to the excellent thermal stability and compactness, the generated ceramic layer was able to keep the barrier effect under prolonged heat or flame attack, which was also the main reason for achieving the long-lasting refractoriness of bi-layered coating. To further make clear the possible reaction of ceramization, the EDS mapping was conducted and thus provided more details. The ceramic layer was mainly composed of ten sorts of elements, but the distribution of elements was obviously distinct. Firstly, six elements were not occupied throughout the ceramic layer. Among them, the elements silicone and magnesium show a similar distribution, and sodium, aluminum, phosphorus, and potassium are distributed in a similar way. As for the remaining four elements, they are spread over almost the whole scanning area. Meanwhile, the specific chemical components of ceramic additives were low melting glass powder (potassium aluminate, sodium aluminate, hydrated aluminum silicate, limestone, silicone dioxide), talc (hydrated magnesium silicate), zinc borate, and aluminum phosphate. Based on the information, the possible reaction process can be speculated. Initially, dehydration copolymerization occurred between sodium aluminate, potassium aluminate, and aluminum phosphate to generate Al/K/Na-metal phosphates. Simultaneously, the resulting multi-metal phosphates were reacted with hydrated magnesium silicate and silicone oxide compounds to form continuous and integral structures. During this process, substances such as zinc borate and calcium oxide serve as cross-linking agents which can undergo a melt polymerization reaction with different metal salts or metal oxides, resulting in the formation of a stable ceramic layer.

## Conclusion

In this work, a bi-layered design was proposed to construct fire retardant coating, which achieved superior efficiency via mere hundreds of microns. Firstly, two silicone rubber formulations with excellent intumescent flame retardancy and ceramization properties were screened, respectively, through extensive formulation optimization. And then, the bi-layered coating was built with intumescent flame-retardant outer layer and ceramic inner layer. Compared with the intumescent flame-retardant single layer and ceramic single layer, the bi-layered coating demonstrated impressive flammability retention and long-lasting barrier effects in response to varying flames or thermal attacks with the same dosage. Specifically, the bi-layered coating, with a thickness of 320 microns, achieved instantaneous self-extinguishing behavior after 10 s of attack by a high intensity flame at 1400 °C and self-extinguishing within 10 s when attacked by a flame at 1200 °C for 60 s. The protected PU foam substrate remained intact and was subject to an extremely low degree of carbonization. The comprehensive fire safety performance of PU foam was also improved, in particular the decreasing PHRR and FGI, and the increasing FPI, TTI, TTP, and residue yield. The bi-layered structure of the coating also demonstrated outstanding long-lasting protection against burn-through applications of metallic material and high-performance glass fabric-reinforced epoxy material. Pure aluminum sheet and GFEP survived a 1400 °C torch attack for only 135 and 173 s, respectively, whereas the coating achieved up to 900 s without burn through for both materials. Further, under fire conditions, the coating resulted in the soft-packed battery maintaining a stable voltage for around 100 s and not short-circuiting for 180 s, whereas the uncoated battery immediately lost its voltage steady state upon contact with fire and short-circuited at 58 s. The mechanism was the bi-layer structure combining the advantages of the rapid response of the intumescent flame-retardant layer and the high stability/low thermal conductivity of the ceramic layer formed by the ceramicisable layer, thus demonstrating excellent results in terms of flame retardancy and long-lasting stabilization of the barrier layer, and the mechanism of intumescent flame retardancy and the possible ceramization reaction process of this system were disclosed. Moreover, the bi-layered design and the optimized formulations are promising in other polymer-based coatings, such as epoxy-based coating. In summary, the present work carried out an ingenious structural design and intensive component optimization to obtain a novel bi-layered coating, which was applicable to a wide range of scenarios.

## Supplementary Information

Below is the link to the electronic supplementary material.

Supplementary file1 (MP4 853 KB)

Supplementary file2 (MP4 8932 KB)

Supplementary file3 (MP4 8986 KB)

Supplementary file4 (MP4 6983 KB)

Supplementary file5 (MP4 3639 KB)

Supplementary file6 (MP4 401 KB)

Supplementary file7 (MP4 9924 KB)

Supplementary file8 (DOCX 19494 KB)
