# Supplementary material for: Bi-Layered, Ultrathin Coating Initiated Relay Response to Impart Superior Fire Resistance for Polymeric and Metallic Substrates
Source: Nanomicro Lett. 2025 Apr 25;17:231. doi: 10.1007/s40820-025-01739-8 (PMC12031706; doi:10.1007/s40820-025-01739-8)
Supplement: Supplementary file 8 — Supplementary file8 (DOCX 19494 KB) [file 40820_2025_1739_MOESM8_ESM.docx]

Supporting Information for

**Bi-Layered, Ultrathin Coating Initiated Relay Response to Impart Superior Fire Resistance for Polymeric and Metallic Substrates**

Wei Tang^1,3^, Qi Chen^1,5^, Junxiao Li^2^, Xiang Ao^1,5^, Yunhuan Liu^1,5^, Lijun Qian^2^*, Silvia González Prolongo^3,6^, Yong Qiu^2^, De-Yi Wang^1,4^*

^1^ IMDEA Materials Institute, C Eric Kandel 2, 28906, Madrid, Spain

^2^ College of Light Industry Science and Engineering, Beijing Technology and Business University, Fucheng Road 11, 100048, Beijing, P. R. China

^3^ Materials Science and Engineering Area, Escuela Superior de Ciencias Experimentales y Tecnología, Universidad Rey Juan Carlos, Calle Tulipán s/n, 28933 Móstoles, Madrid, Spain

^4^ Escuela Politécnica Superior, Universidad Francisco de Vitoria, Ctra. Pozuelo-Majadahonda Km 1, 800, 28223, Pozuelo de Alarcón, Madrid, Spain

^5^ E.T.S. de Ingenieros de Caminos, Universidad Politécnica de Madrid, Calle Profesor Aranguren 3, 28040 Madrid, Spain

^6^ Instituto de Investigación de Tecnologías para la Sostenibilidad. Universidad Rey Juan Carlos

*Corresponding authors. E-mail: [qianlj@th.btbu.edu.cn](mailto:qianlj@th.btbu.edu.cn) (Lijun Qian); [deyi.wang@imdea.org](mailto:deyi.wang@imdea.org) (De-Yi Wang)

**S1 Detailed Information for Some Raw Materials**

Main ingredients in ADDV-10 Component A: Dimethylpolysiloxane (50%), Silicium dioxide (40%), Dimethicone (9.9%), Silicic acid ethyl ester (0.1%).

Main ingredients in ADDV-10 Component B: Dimethylpolysiloxane (45 - < 50%), Silicium dioxide (35 - < 40%), Polymethylhydrosiloxane (10 - < 15%), Dimethicone (1 - < 5%), Silicic acid ethyl ester (< 0.1%).

Aluminum plate was treated with a primer provided by Dowsil (1200OS).

**S2 Detailed Description for Ceramifiable System**

The morphology and size of additives in ceramifiable system are shown in Fig. S1.


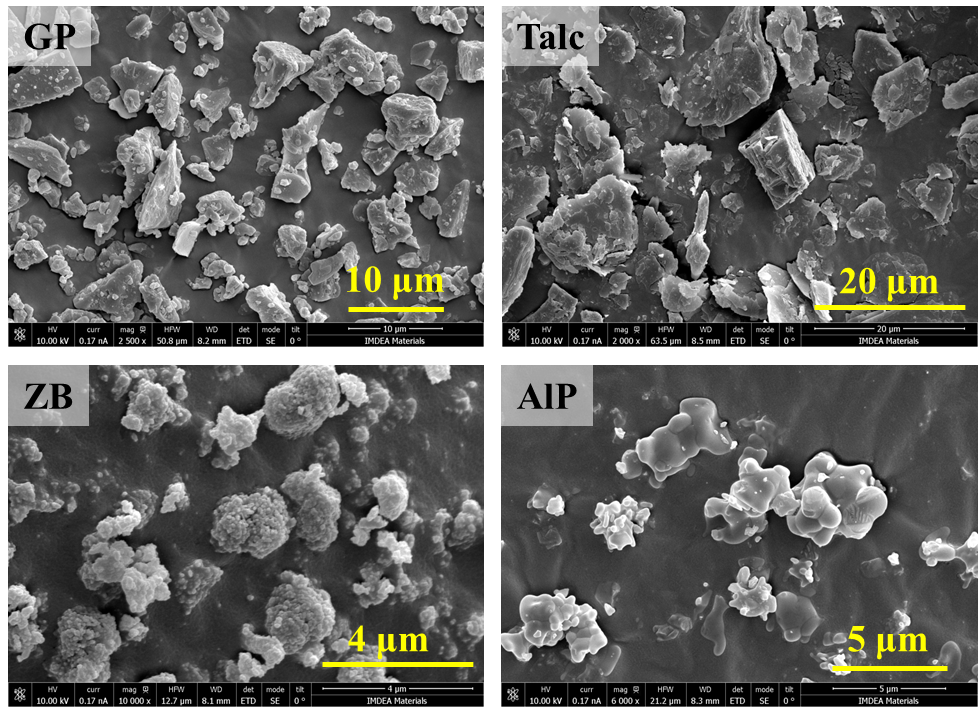


**Fig.** **S1** The micromorphology of ceramifiable additives

In the ceramization reaction, low-melting glass powder, talc, zinc borate, and aluminum phosphate play distinct roles in promoting the formation of ceramic phases and enhancing the high-temperature resistance of materials:

- Low-Melting Glass Powder (GP): Primarily functions to reduce the ceramization temperature, facilitating the early formation of ceramic phases; Upon exposure to high temperatures, it melts and forms a glassy phase, improving material fluidity and promoting the bonding of solid-phase particles, thereby enhancing the density and mechanical strength of the ceramic layer.
- Talc: Acts as a filler and flux, decomposed at high temperatures to form magnesium aluminum silicate structures, which contribute to the formation of a heat-resistant ceramic layer. It also improves the mechanical strength and thermal stability of the ceramicised layer.
- Zinc Borate (ZB): Serves as a flux, lowering the sintering temperature and accelerating the ceramization process; It participates in the formation of the glassy phase, enhancing fire resistance while improving the adhesion and crack resistance of the ceramic layer.
- Aluminum Phosphate (AlP): Functions as a high-temperature binder, reacting with other components at elevated temperatures to form a stable ceramic network structure.

In summary, these components interact synergistically to facilitate rapid ceramization under high-temperature conditions, thereby improving fire resistance and mechanical performance.

**S3 Preparation of Polymer Materials and Battery for Substrates**

Fabrication of rigid polyurethane foam (PU): H200-AT components A and B were homogeneously mixed in the mass ratio of 1:1 and stirred for 40 s at a speed of 1000 rpm. The mixture was then poured into a mold and aged at room temperature for 24 h to obtain PU foam.

Fabrication of glass fabric reinforced epoxy resin composites (GFEP): Ten layers of dry glass fabric were stacked upon each other by the notation [0/90)2]T for GFEP. The epoxy resin and curing agent with stoichiometric ratio was mixed evenly and degassed under vacuum for 10 mins. The details for fabricating the laminate can be found in our former publication [X. Ao, J. Xiao, J. Hobson, J. de la Vega, G. Yin et al., Bilayer coating strategy for glass fiber reinforced polymer composites toward superior fire safety and post-fire mechanical properties, Composites Communications **44** (2023) 101763]. After the infusion process, the target material was obtained after heating at 50 ℃ for 3 h and 100 ℃ for 3 h. The average thickness of the resulting laminates was 2.4 mm.

Preparation of battery: As shown in Fig. S2, the components of all-solid-state soft-package batteries of LFP|SPEs| Li, including LFP positive and Li negative electrodes, and PEO-based solid polymer electrolytes, as separators simultaneously. Firstly, the tabs were adhered onto the surface of the Li anode and the current collector of the LFP cathode. Then the PEO-based SPE was used to cover the LFP cathode surface and edges to ensure the complete separation between the LFP cathode and Li anode. After this, put the Li anode on the other side of the SPE. All the components were enclosed in an aluminum-coated plastic film. The soft-package lithium battery consists of a LiFePO_4_ cathode and a Li anode both in size of 3×3 cm^2^, and a modified PEO-based solid polymer electrolyte of 5×5 cm^2^. Only two tabs were exposed to the outside for connection to an external circuit for monitoring electrochemical parameters, such as voltage and current. All the processes were carried out inside of glove box filled with argon atmosphere.


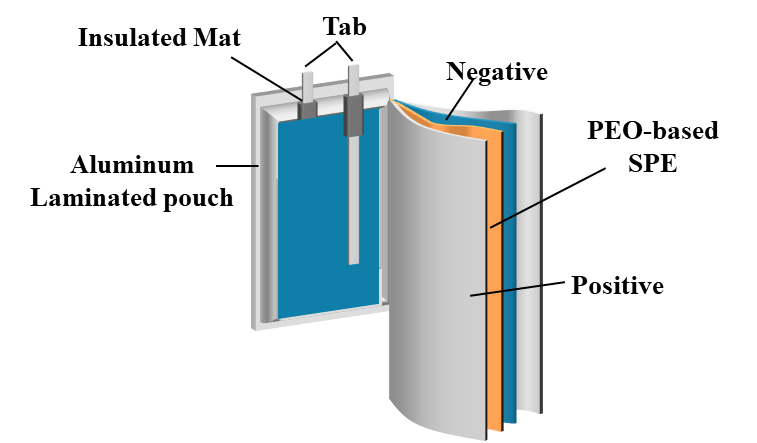


**Fig. S2** The schematic diagram of soft package battery

**S4 Explanation for Difference in OCV Values of Two Batteries**

The voltage of a lithium-ion battery is determined by the electrode potential. Voltage, also known as potential difference, is a physical quantity that measures the energy difference of electric charges in an electrostatic field due to different potentials. The electrode potential of lithium-ion batteries is about 3V, and the voltage of lithium-ion batteries varies with different materials. For example, a general lithium-ion battery has a nominal voltage of 3.7 V and a full-charge voltage of 4.2 V. A lithium iron phosphate (LiFePO4, LFP) battery has a nominal voltage of 3.2 V and a full-charge voltage of 3.65 V. In other words, the potential difference between the positive electrode and the negative electrode of a lithium-ion battery in practical use cannot exceed 4.2 V, which is a requirement based on material and use safety.

In the system of lithium metal batteries (Li as anode material), the voltage range of 2~3 V around is at a normal level. This is because the OCV was generally influenced by the degree of smoothness, the density of the LFP cathode, and the degree of contact between the components of the LFP cathode, Li anode, and PEO-based SPE. The OCV will increase first and keep a steady state during the resting process before any tests if without any possibility of a micro short circuit (voltage<2 V). Therefore, in this work, the different OCV for two batteries was in an acceptable normal range.

**S5 Fabrication of Silicone Rubber Composites**

The raw materials were mixed evenly according to the designated ratios by stirring for 10 minutes. And then 12 g mixture for each sample was transferred into a cylindrical mold with a 48 mm diameter. Finally, silicone rubber composites with the thickness of 7.5 mm can be obtained after the curing for 1 hour at 50 ℃. It should be mentioned that the IFR system was composed of piperazine pyrophosphate and melamine polyphosphate with a mass ratio of 7:3.

**S6 Application of Coating**

Firstly, the uniform mixture was obtained by mixing ceramic fillers and silicone rubber, and then coated on the substrate via a blade. Afterwards, the sample attached with a single layer was put into an oven with 50 ℃ for the precuring stage. Meanwhile, the intumescent flame retardant / synergist additives were dispersed in silicone rubber prepolymer. The blade coating method was employed again for applying the IFR layer on the ceramic layer. Finally, the bi-layered coating was successfully fabricated after the curing in an oven with 50 ℃ for 1 hour. The reference samples which were applied with only one layer coating were also constructed.

As shown in Fig. S3, the ceramifiable layer contains metal elements such as aluminum (Al), sodium (Na), zinc (Zn), and potassium (K), whereas the outer IFR layer contains only aluminum metal element. As observed in the EDS mapping, the inner layer exhibits the presence of these elements, while the upper layer contains only aluminum metal element. Furthermore, the EDS mapping of aluminum reveals a stronger signal in the inner layer, which can be attributed to the higher aluminum content in the ceramifiable layer. This is due to the presence of aluminum in both the low-melting glass powder and aluminum phosphate, whereas in the surface IFR layer, aluminum is present only in aluminum oxide. Based on the analysis, it is confirmed that the bi-layered structure was constructed in this work.


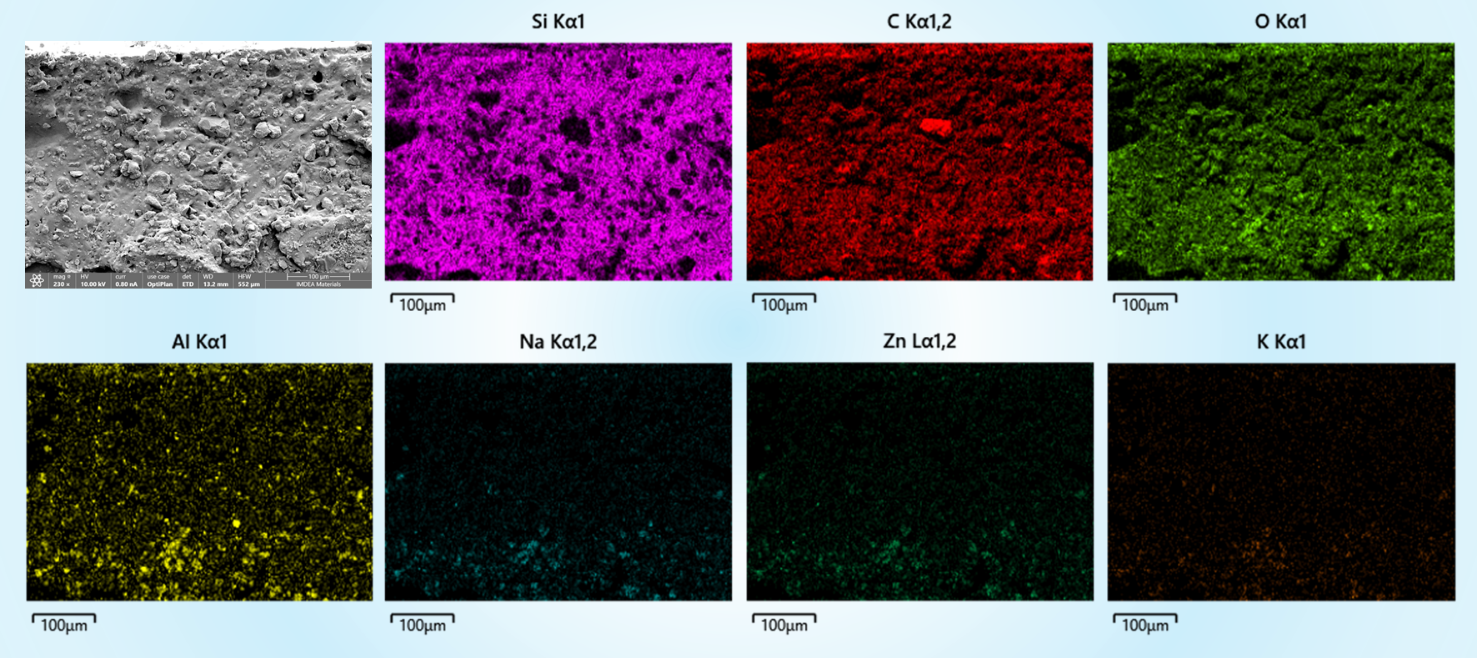


**Fig.** **S3** EDS mapping of typical elements for bi-layered coating


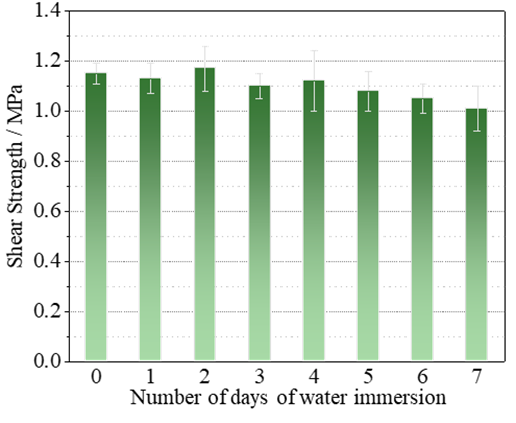


**Fig.** **S4** The shear strength of inner ceramifiable coating after the water immersion treatment

**Table S1** The tensile strength and elongation at break of silicone rubber composites

|  | Tensile Strength / MPa | | | Elongation at break / % | | |
| --- | --- | --- | --- | --- | --- | --- |
|  | RT | 50 ℃ | 80 ℃ | RT | 50 ℃ | 80 ℃ |
| SiR | 3.12 | 2.90 | 2.53 | > 600 | > 600 | > 600 |
| 2Al_2_O_3_/38IFR/SiR | 1.58 | 1.55 | 1.26 | 109 | 126 | 129 |
| 30_G_2_T_6_Z_2_A_/SiR | 1.62 | 1.63 | 1.50 | 75 | 83 | 102 |

**S7 Characterization**

Limiting oxygen index of samples with the dimensions of 130.0 mm × 6.5 mm × 3.2 mm was tested by the facility (Fire Testing Technology, UK) based on the ASTM D2863 standard. The UL 94 vertical burning test was conducted on a chamber (Fire Testing Technology, UK) according to ASTM D3801 standard with the sample dimensions of 125.0 mm × 12.7 mm × 3.2 mm. The fire resistance of PU foams was evaluated by two fire attack tests, as displayed in Fig. S5. The dimensions of PU foams are 100.0 mm × 100.0 mm ×30.0 mm. The comprehensive fire-safety performance was provided by FTT equipment (Fire Testing Technology, UK) based on ISO 5660-1 standard with a heat flux of 50 kW/m^2^. The dimensions of coated PU and GFEP samples are 100.0 mm × 100.0 mm ×30.0 mm and 100.0 mm × 100.0 mm ×2.4 mm, respectively. The burn through resistance was carried out according to the approach of Fig. S6 and UL 2596 standard. The thermal imager (AT31U1Z, IRay Technology) was used to record the temperature.

The thermal behavior was analyzed by Q50 (TA Instruments) thermogravimetric instruments with the heating speed of 20 ℃/min. Meanwhile, the iS50 FTIR spectroscopy (Nicolet) was connected to the TGA facility to detect the structural information of pyrolysis fragments. The coating and residues were observed by the Apreo 2S LoVac scanning electron microscope (Thermo Fisher Scientific, FEG-SEM) and SZX10 microscope (Olympus). Besides, the EDS detector was utilized to provide elemental mapping. The pyrolysis behavior of flame retardant was explored by the EGA/PY-3030D multi-shot pyrolyzer (Frontier Lab) and Clarus 680/SQ 8T Chromatography-mass spectrometer (PerkinElmer, GC/MS) with the pyrolysis temperature of 500 ℃.

Fire resistance and barrier effect of the fabricated silicone rubber composites were evaluated with a new method which was designed and established by our lab. The sample was attacked by the strong torch fire with around 1400 ℃ for 120 s, during which the temperature of the sample backside was recorded in real time via the thermocouple. Additionally, the burning time of sample after removing the torch was also recorded. Each sample before and after the test was also weighed to calculate the residue rate.

In Fig. S5, the application time is 10 s for the strong torch fire and 60 s for the weaker UL 94 fire, respectively.


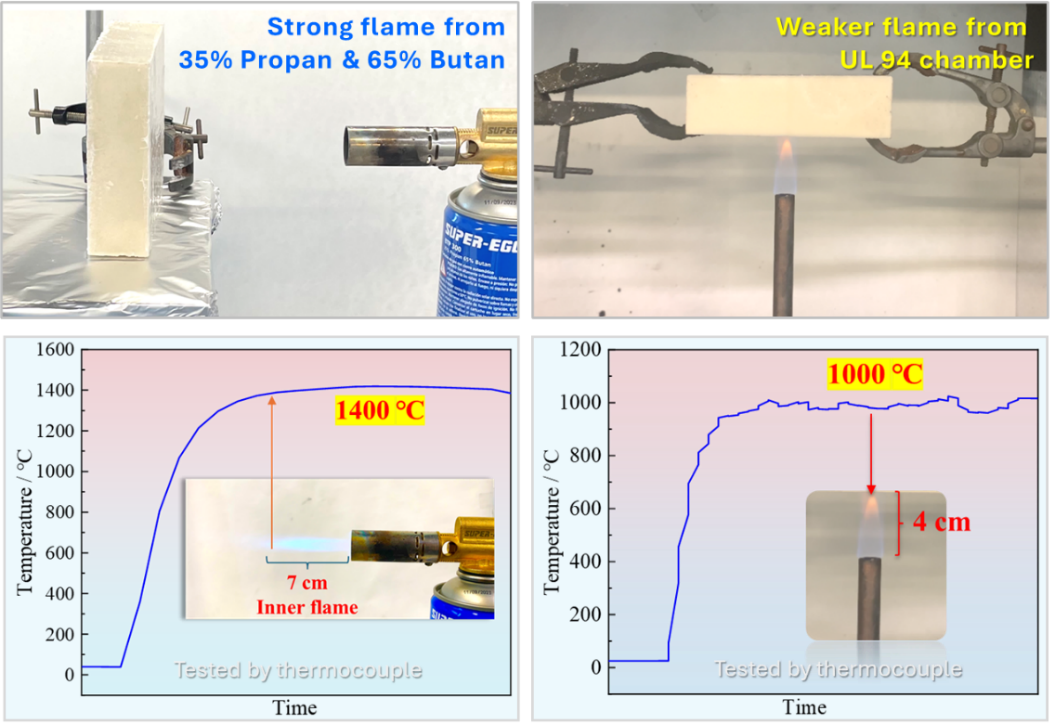


**Fig.** **S5** The measurement methodology of fire retardancy for PU foams


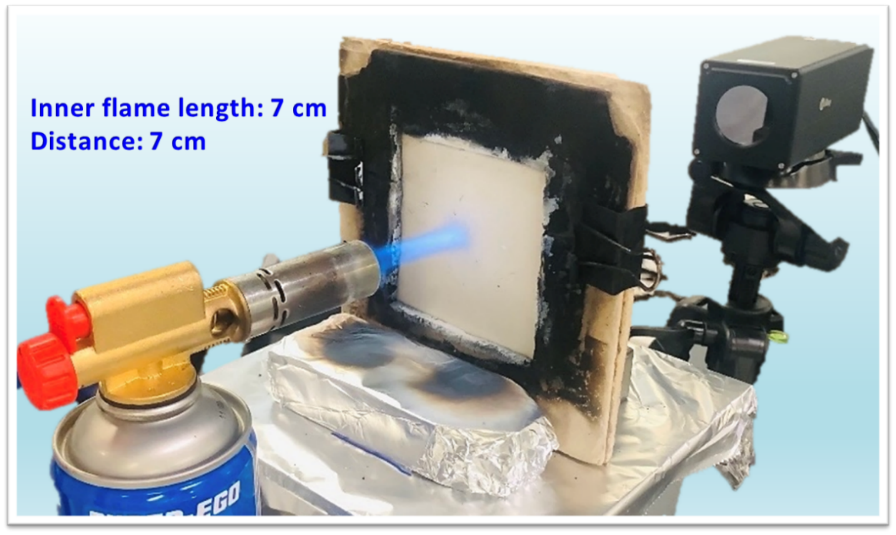


**Fig. S6** The measurement method for assessing the burn-through resistance

**S8 Optimization of Ceramic and IFR Formulations**

The excellent flame retardancy of 2Al_2_O_3_/38IFR was contributed by both condensed-phase and gas-phase effects. Through comparing the combustion of Fig. S7(a) and (b), the self-extinguishing phenomenon aroused resulted from the IFR/Al_2_O_3_ system, while neat silicone rubber kept burning for a much longer time. The reason was deduced that some flame retardant fragments were generated from polyphosphate components to except quenching and dilution effect. Except for the gas-phase part, it was distinct that an integrated and compact char residue was formed after the attack of fire, while there were many cracks on the surface of neat silicone rubber and the residue was also easy to separate from the substrate. Therefore, it can be concluded that the IFR/Al_2_O_3_ were able to interact with silicone rubber during combustion. Furthermore, IFR/Al_2_O_3_ endowed silicone rubber with UL 94 V-0 rating in Fig. S8(a) and over 27% of LOI, which indicated its good nonflammability.


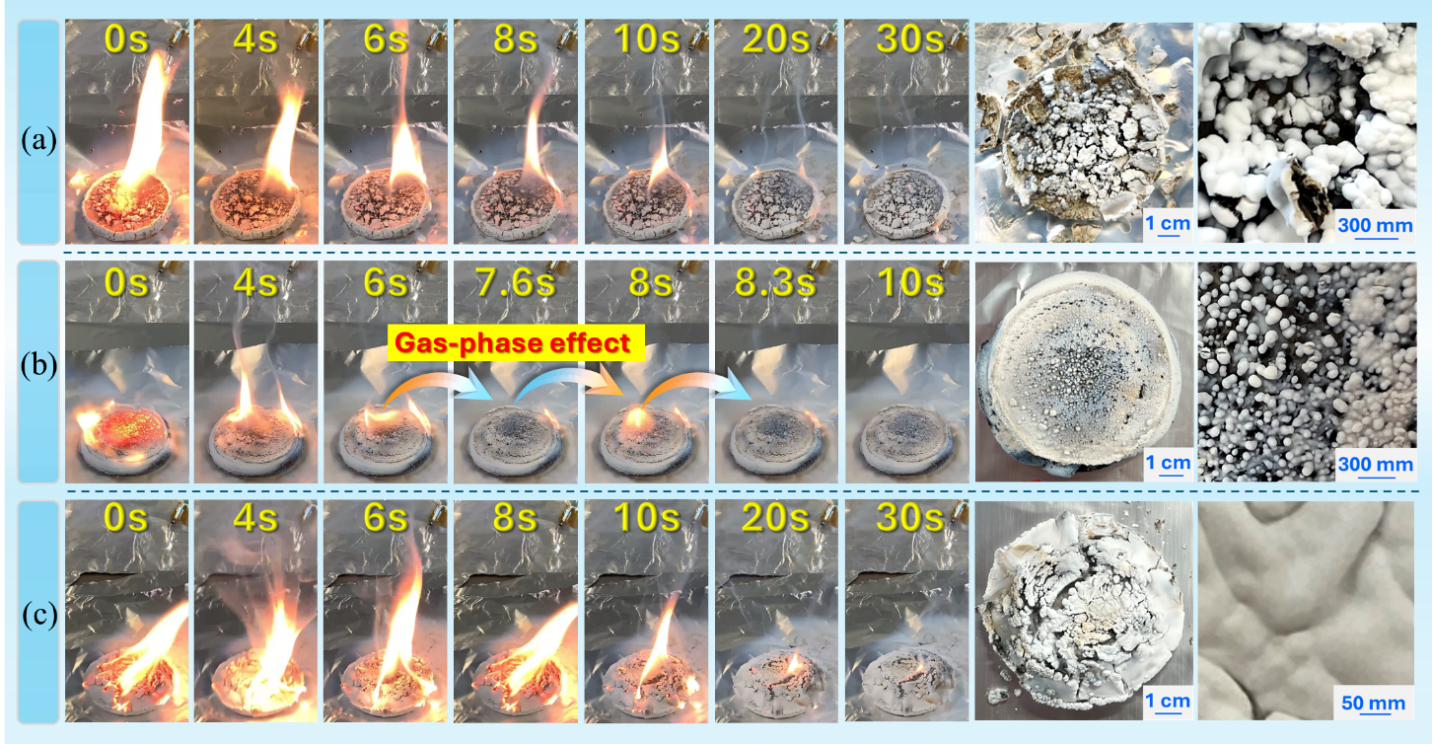


**Fig.** **S7** The burning process and morphology after direct fire damage tests of neat silicone rubber (**a**), silicone rubber containing 2Al_2_O_3_/38IFR (**b**), and silicone rubber containing 30_G_2_T_6_Z_2_A_ (**c**)

As to the burning time, silicone rubbers with 10_G_5_T_20_Z_5_A_ and 10_G_5_T_5_Z_20_A_ systems exhibited 28 and 26 s of afterflame, respectively, which were lower than other samples. Drawing on these results, it can be summarized that glass powder is beneficial in yielding a layer with excellent barrier property, whereas systems containing borates and phosphates are more effective in suppressing flames. For the bi-layered coating structure in this work, the ceramic layer was attached between the substrate and IFR layer. Hence, the ceramifiable silicone rubber did not contact the fire directly in the application. Owing to the real case, the better barrier effect of ceramic layer was more essential for the efficacy of bi-layered coating. Based on the above concerns, formula 30_G_2_T_6_Z_2_A_ was more suitable for fabricating the ceramic coating.

IFR/Al_2_O_3_ endowed silicone rubber with UL 94 V-0 rating in Fig. S8(a) and over 27% of LOI, which indicated its good nonflammability. As displayed in Fig. S8(b), even though there was no UL 94 flame retardant rating for the silicone rubber containing 30_G_2_T_6_Z_2_A_, the damaged length was essentially negligible due to the low combustion intensity. Notably, the ceramic layer was gradually formed during the burning process after the first fire application, and then prevented the matrix from being destroyed in the second fire application. In other words, once a ceramic layer was created, the materials cannot be ignited anymore. The resulting ceramic layer is therefore an excellent candidate for insulation material. Meanwhile, the LOI of 30_G_2_T_6_Z_2_A_/silicone rubber reached above 27%, indicating the refractory level.


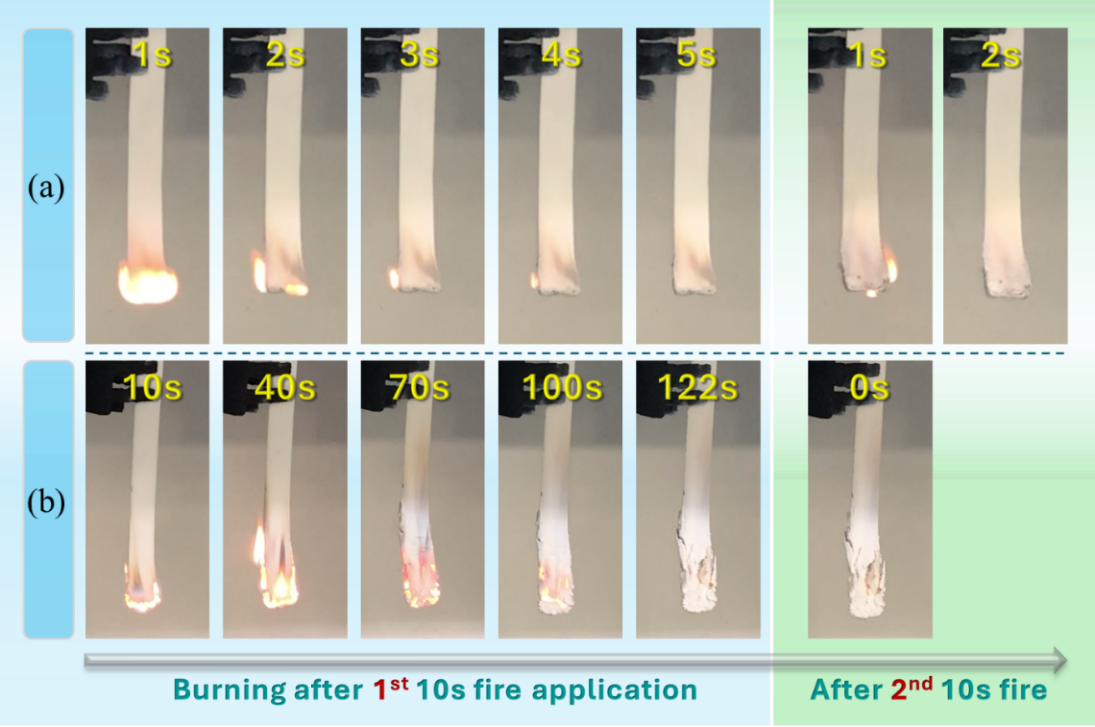


**Fig.** **S8** The burning behavior of silicone rubber containing 2Al_2_O_3_/38IFR (**a**), and silicone rubber containing 30G2T6Z2A (**b**) in the UL 94 test

**S9 Main Mechanism of Intumescent Flame Retardant in This Work**

Intumescent flame retardants function by forming protective, insulating char layers when exposed to heat or flame, effectively reducing heat transfer and inhibiting further combustion. The mechanism typically involves three key components: an acid source, a carbon source, and a blowing agent. Upon heating, the acid source (pyrophosphate structure) decomposes to produce phosphoric acid, which catalyzes the dehydration of the carbon source (piperazine structure) and part of matrix structures, leading to char formation. Simultaneously, the blowing agent (melamine structure) releases non-flammable gases, causing the char layer to expand into a porous, foamed structure. This expanded char acts as a thermal and oxygen barrier, slowing down heat penetration, reducing flammable gas release, and ultimately enhancing the flame-retardant efficiency of the material.

**S10 Fire Damage Test on PU Foam with a Fire of 1000 ℃**

To thoroughly evaluate the efficacy and benefit of the bi-layered coating, the built samples were characterized under another different fire condition with the temperature of around 1000 ℃. The test process and burning situation are detailed in Fig. S9(a-d) and Video 3. Similarly, the PU without coating was extremely easy to be ignited, and burned with a strong intensity and rapid propagation behavior until the substrate was completely carbonized, which can be observed in Fig. S9(a, e). In comparison, the bi-layered coating enabled PU excellent flame retardancy and no fire spread, as shown in Fig. S9(b). Especially, even though the fire application time was up to 60 s, the self-extinguishing time decreased to mere 10 s from the 237 s of neat PU, and the formed barrier layer retained its shape stability and denseness during the whole process in Fig. S9 (f) and Fig. S10, which all implied that the matrix under the coating was not ignited. In addition, the morphology of PU matrix in Fig. S9(i) exhibited that there was a mild thermal aging with the matrix after the fire destroy for 60 s, demonstrating the superior barrier effect of the char layers by bi-layered coating, especially the thermal insulation ability. As to the other two reference samples in Fig. S9(c, d), the heavier combustion intensity and longer self-extinguishing time illustrated that the single layer was not able to produce the barrier layers with comprehensive protection effect. Specifically, the single ceramic coating cannot form the effective ceramic residue layer shortly, resulting in a high self-extinguishing time of 27 s. But the ceramic residue layer from single ceramifiable coating still performed good thermal insulation effect, which can be proved by the matrix morphology in Fig. S9(j). PU with the single IFR layer was able to exert good nonflammability, and the coating was not ignited fully during the fire attack process in Fig. S9(d). The self-extinguishing time was 14 s, which was within the acceptable range. But for the IFR single layer, the problem was that the generated char layers were not thermally stable enough, especially under the long-term fire attack. Hence, the matrix covered by the IFR coating was seriously carbonized during the test, as displayed in Fig. S9(k). On top of these, a conclusion can be given that the bi-layered strategy was able to achieve the outstanding barrier effect and flame retardancy with hundreds of microns.


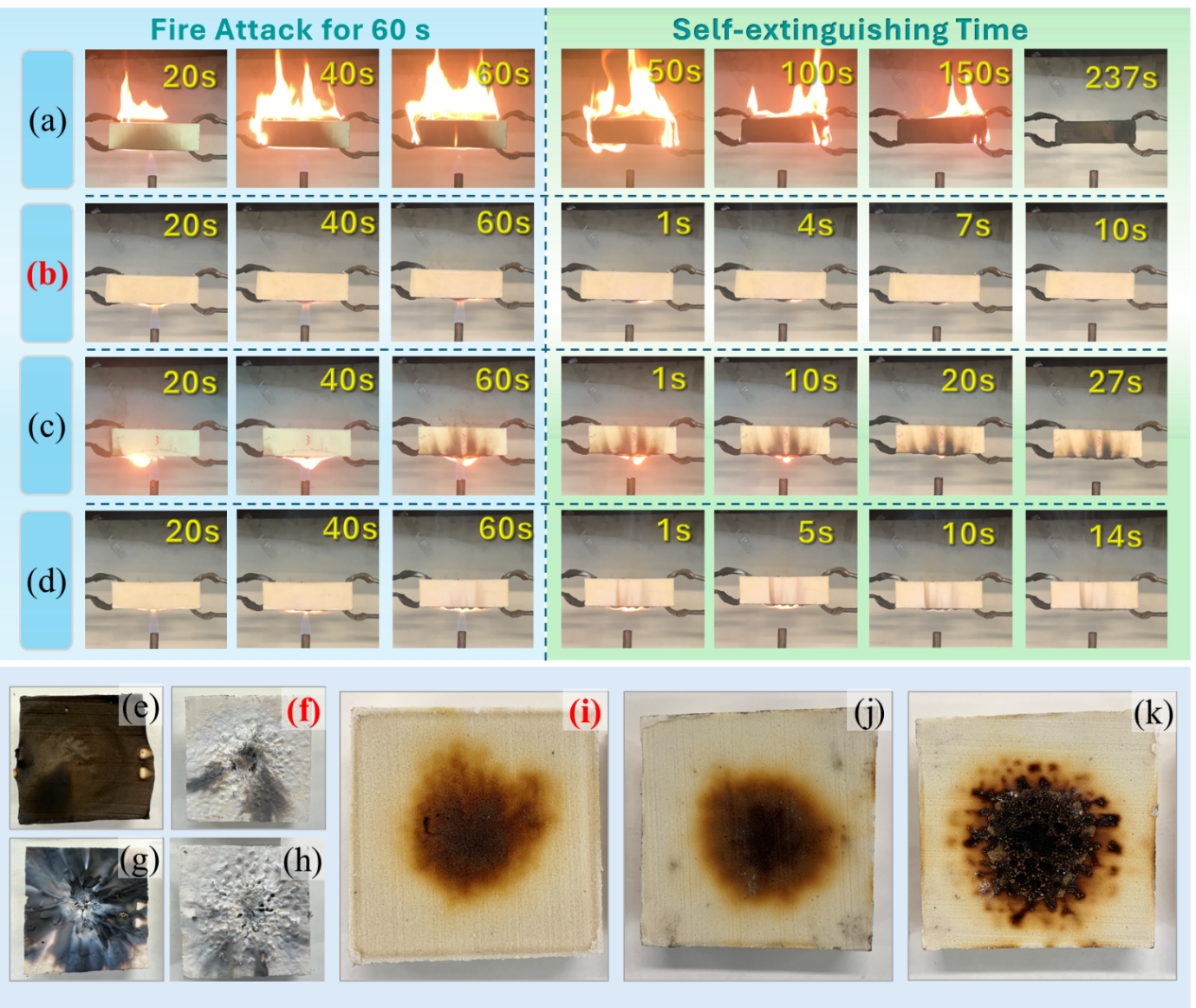


**Fig.** **S9** The test process (**a-d**), coating (**f-h**) & substrate (**e, i-k**) morphology after fire damage for neat PU (**a, e**), PU with bi-layered coating (**b, f, i**), PU with ceramic single layer (**c, g, j**), and PU with IFR single layer (**d, h, k**)


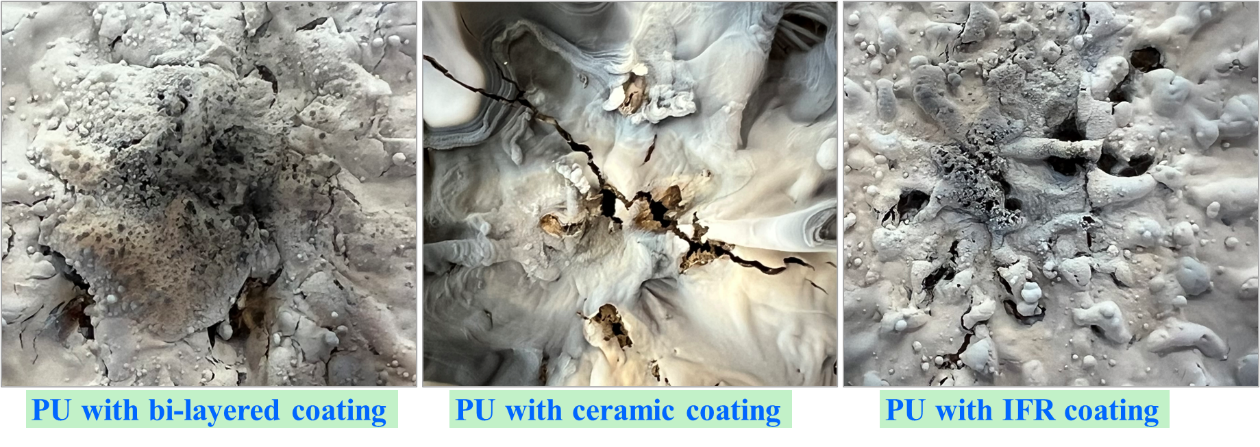


**Fig.** **S10** The morphology of char layers after fire test of 1000 ℃ for 60 s


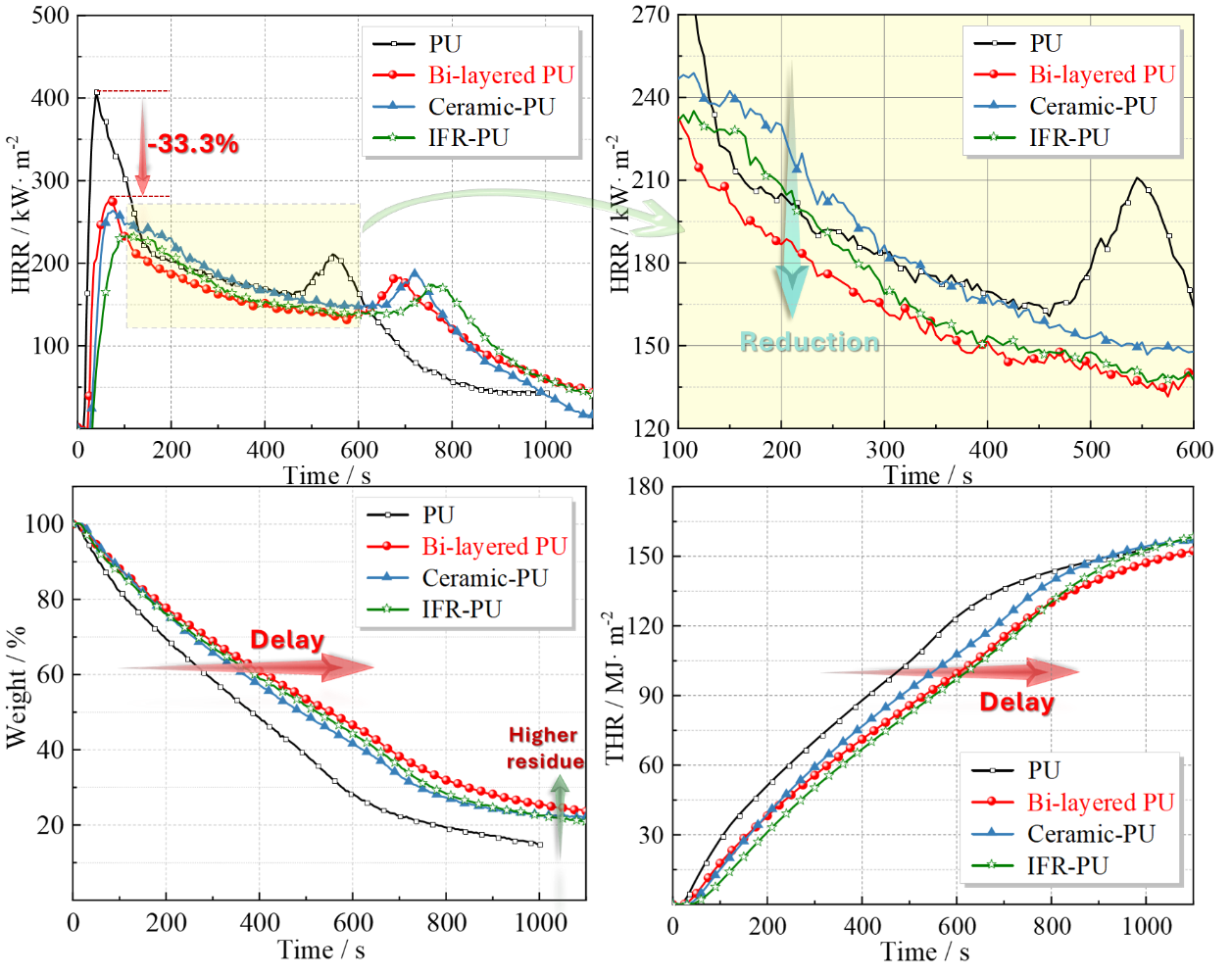


**Fig.** **S11** The HRR, weight loss and THR curves of PU with different coatings


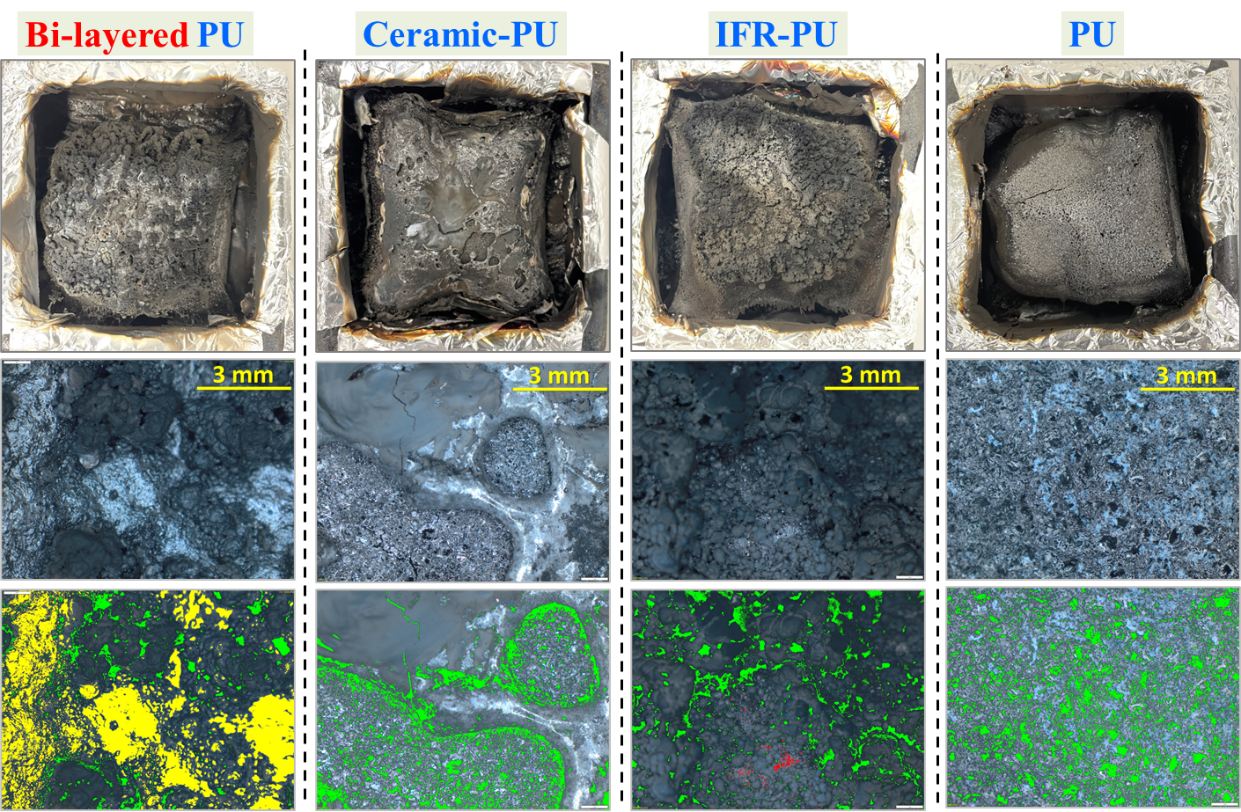


**Fig.** **S12** The surficial char morphology of PU samples after cone calorimeter test


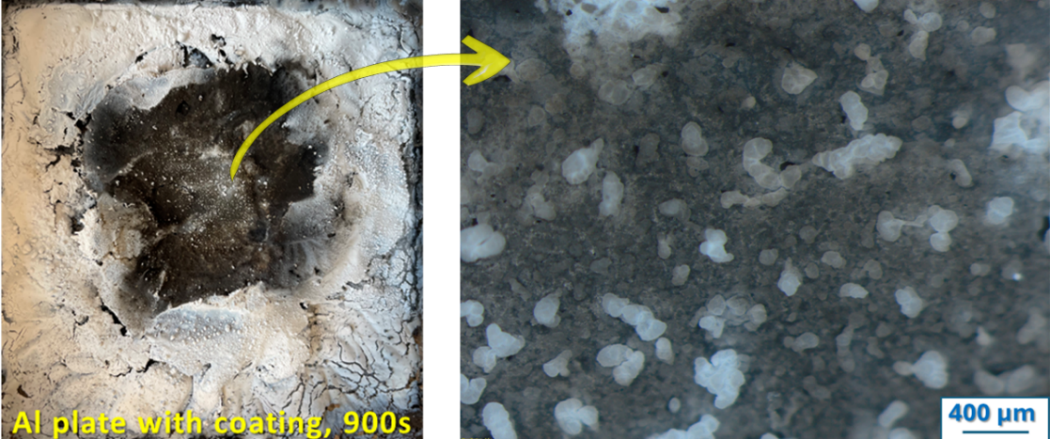


**Fig.** **S13** The micro morphology of char after burn-through resistance for aluminum plate


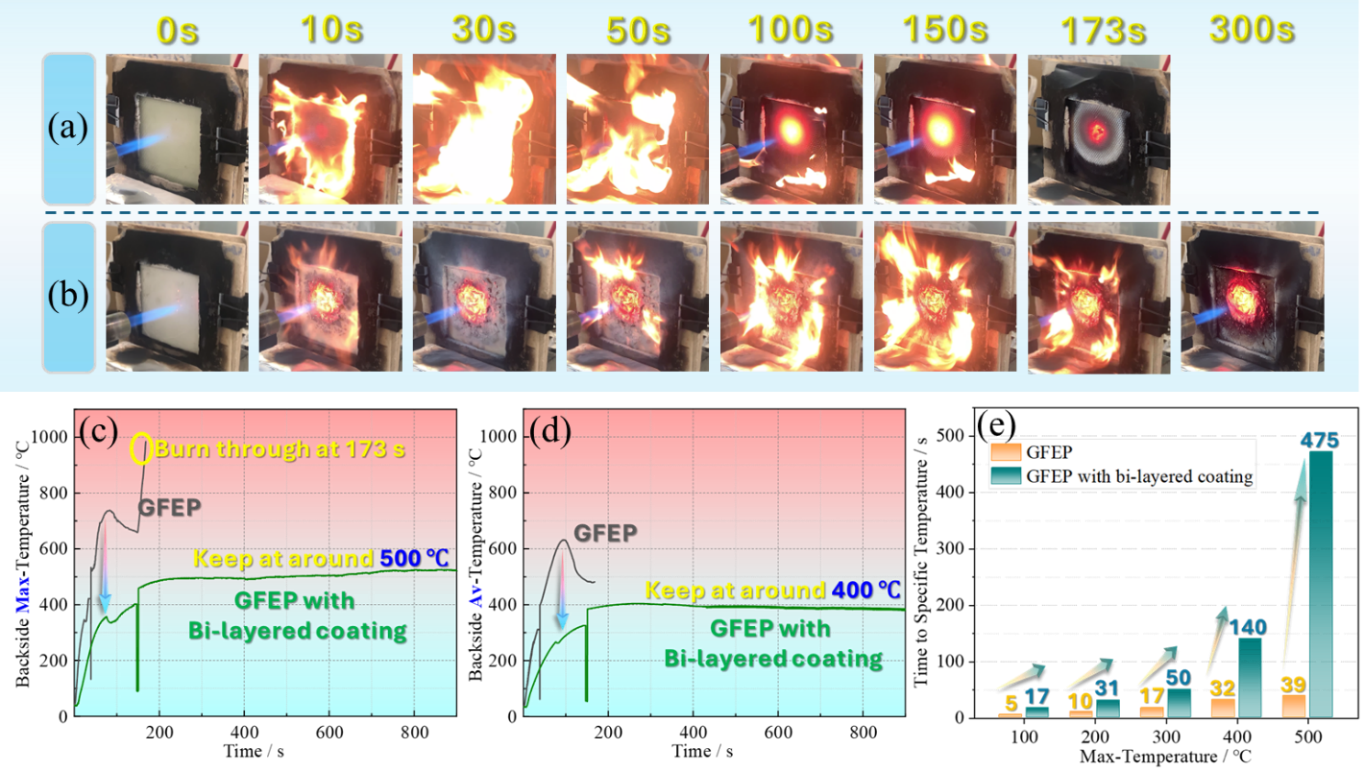


**Fig.** **S14** The initial stage of burn through test for GFEP (**a**) and GFEP with bi-layered coating (**b**), the maximum (**c**) and average (**d**) temperature change curves of backside with the attack time, and the time to specific temperature of backside (**e**)


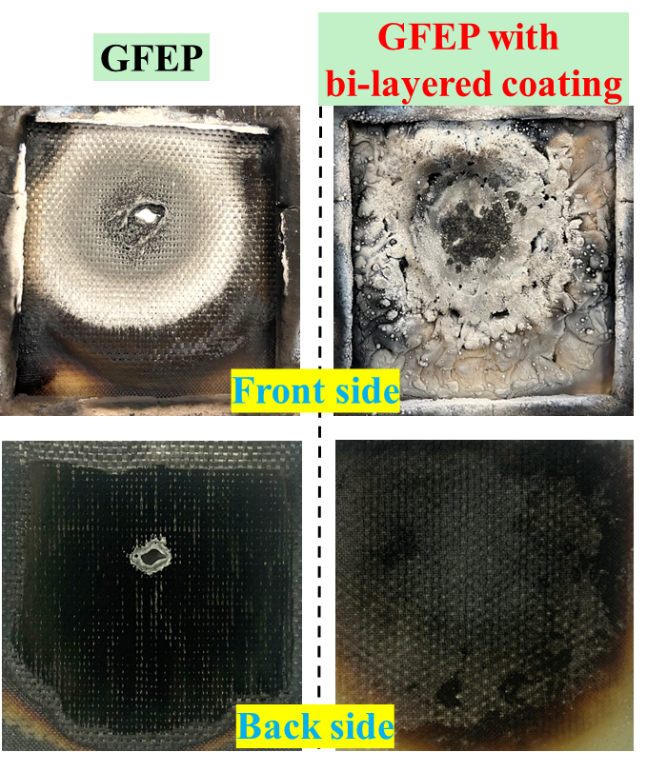


**Fig.** **S15** The morphology of GFEP after burn-through resistance


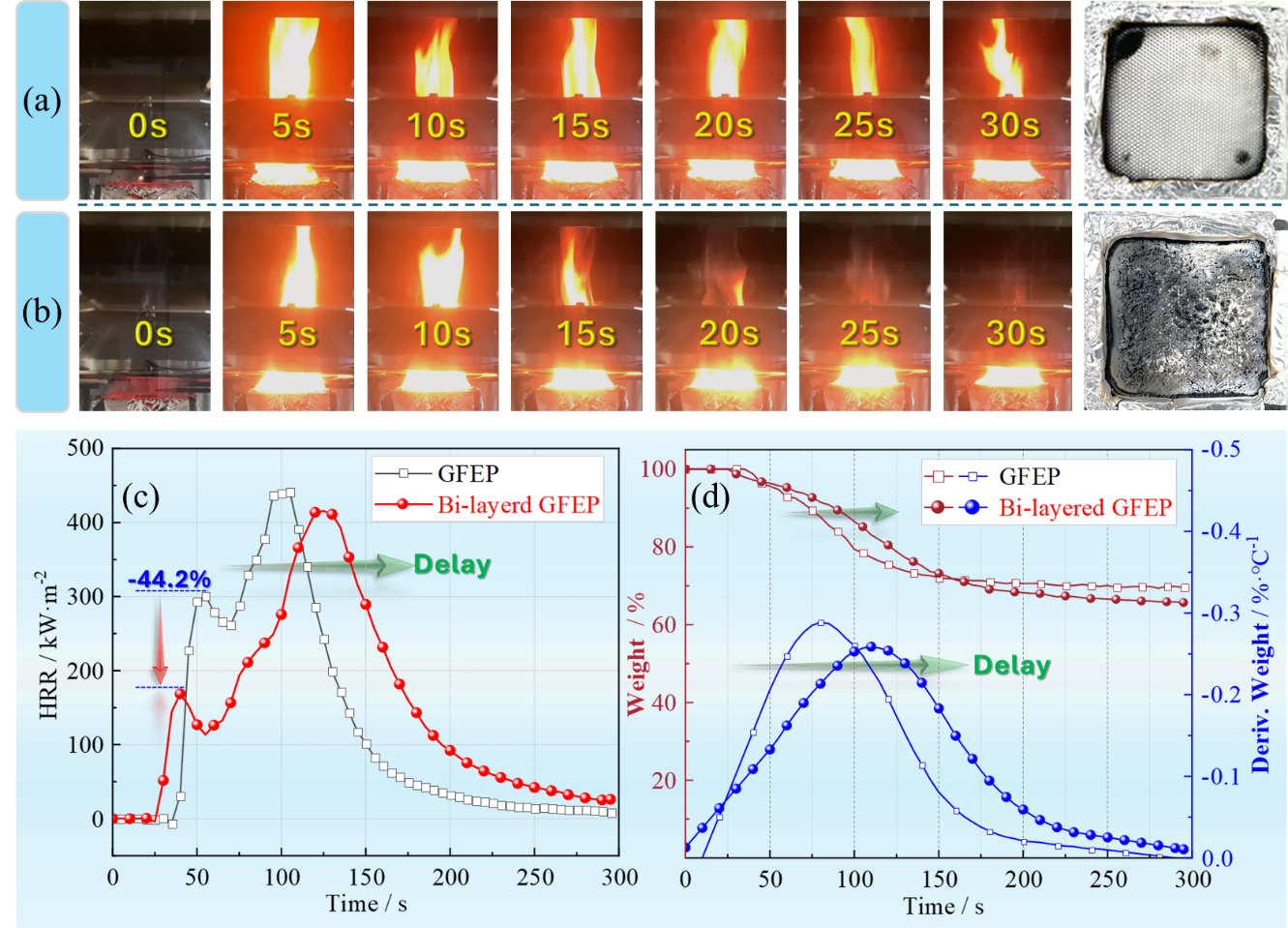


**Fig.** **S16** The initial burning conditions of GFEP (**a**) and GFEP with bi-layered coating (**b**), the HRR curves (**c**), and the wight loss curves (**d**)

**Table S2** The typical parameters of PU samples from cone calorimeter tests

| Sample | TTI  / s | TTP  / s | PHRR  / kW·m^-2^ | THR  / MJ·m^-2^ | TSR  / m^2^·m^-2^ | R_end_  / % | PMLR  / g·s^-1^ | Av-EHC  / MJ·kg^-1^ |
| --- | --- | --- | --- | --- | --- | --- | --- | --- |
| PU | 3±0 | 40±0 | 405±4 | 151.4±1.6 | 3770±104 | 16.0±1.0 | 0.202±0.010 | 23.4±0.1 |
| Bi-layered PU | 13±0 | 75±5 | 270±10 | 151.5±0.8 | 4010±120 | 24.5±1.1 | 0.130±0.008 | 23.2±0.2 |
| Ceramic-PU | 13±0 | 75±5 | 270±7 | 156.0±1.0 | 4712±190 | 21.3±0.8 | 0.157±0.009 | 23.4±0.2 |
| IFR-PU | 13±0 | 110±5 | 230±5 | 163.0±1.9 | 4510±202 | 21.2±1.7 | 0.209±0.010 | 22.8±0.1 |

**Table S3** The typical parameters of GFEP samples from cone calorimeter tests

| Sample | TTP  / s | TTI  / s | PHRR-1  / kW·m^-2^ | PHRR  / kW·m^-2^ | THR  / MJ·m^-2^ | TSR  /·m^2^·m^-2^ | R_end_  / % | PMLR  / g·s^-1^ | Av-EHC  / MJ·kg^-1^ |
| --- | --- | --- | --- | --- | --- | --- | --- | --- | --- |
| GFEP | 105±0 | 31±0 | 301±12 | 441±20 | 35.9±1.6 | 1277±56 | 69.6±0.2 | 0.203±0.012 | 25.6±1.1 |
| Bi-layered GFEP | 125±5 | 21±0 | 168±15 | 415±12 | 43.9±1.9 | 2082±108 | 65.5±0.3 | 0.165±0.010 | 24.1±1.3 |

**S11 The low heat transfer efficiency of the intumescent char layer and ceramic char layer during burning can be attributed to the following factors**

- Porous and Expanded Structure (Intumescent Char Layer)

The intumescent char layer forms as a result of the thermal decomposition of flame-retardant additives, leading to the release of non-flammable gases that create a highly porous and foamed structure. This porous structure introduces a large number of air pockets, which act as thermal barriers since air is a poor heat conductor. The expanded char layer increases the distance between the heat source and the underlying substrate, slowing down heat transfer.

- Low Thermal Conductivity of Carbonaceous Residue (Intumescent Char Layer)

The intumescent char layer is primarily composed of carbonaceous materials, which inherently have low thermal conductivity. The presence of amorphous carbon and graphitic structures with disordered arrangements further reduces heat conduction.

- Dense and Compact Microstructure (Ceramic Char Layer)

The ceramic char layer forms through high-temperature reactions, leading to the creation of an inorganic, compact, and thermally stable structure. Unlike the intumescent layer, which is porous, the ceramic layer has a denser microstructure that provides superior thermal shielding. The low thermal conductivity of ceramics further minimizes heat transfer.

- Synergistic Heat Barrier Effect (Dual-Layer Structure)

The combination of the intumescent char layer (porous and insulating) and the ceramic char layer (dense and heat-resistant) creates a multi-functional thermal barrier. The outer intumescent layer slows down the initial heat penetration, while the inner ceramic layer prevents further heat propagation, effectively protecting the substrate.

- Radiative and Convective Heat Blocking

The foamed intumescent char traps radiant heat and prevents direct heat flux transmission to the substrate. The ceramic char layer reflects a portion of the heat radiation due to its high-temperature stability and emissivity properties. The porous nature of the intumescent layer reduces convective heat transfer by limiting the movement of hot gases within the material.

In summary, the low heat transfer efficiency of these layers results from their structural characteristics, material composition, and synergistic effects, making the bi-layered coating highly effective in thermal insulation.

**S12 Widen the bi-layered design and flame retardant additives formulation of this work by using EP matrix**

Epoxy resins are one of the most versatile binder candidates for coatings due to their excellent adhesion, stability and cost effectiveness. The bi-layered coating structure and flame retardant formulation designed in this work were also extended to the construction of epoxy-based (EP, Resoltech 1800^®^) coatings. The relevant information for the EP-based coating was shown in **Fig.** S17. And the substrate was aluminum plates for assessing the burn-through resistance.


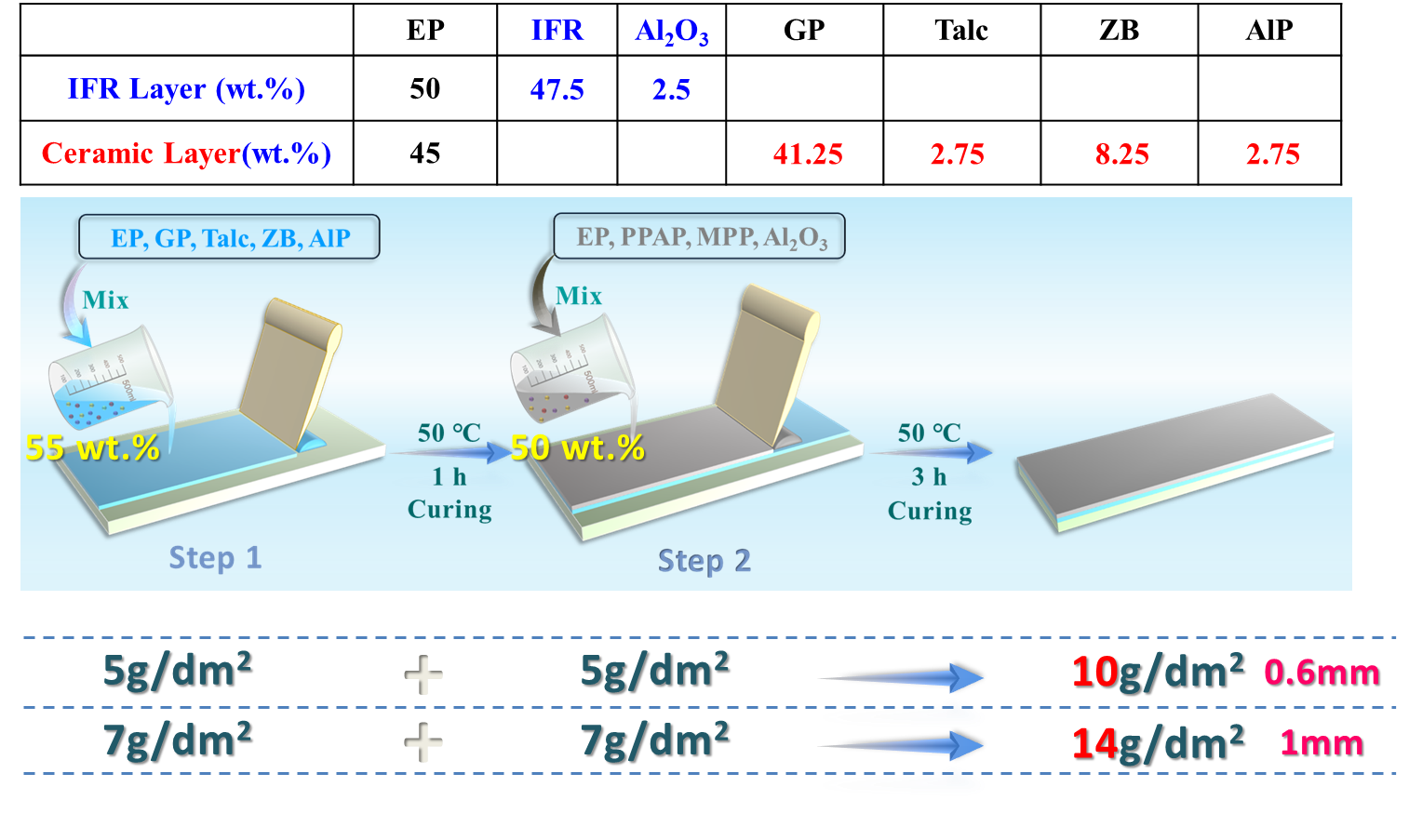


**Fig.** **S17** The formulation, process, dosage and thickness of EP-based coating

The main results are displayed in **Fig.** S18. The 14g/dm^2^ of dosage enabled the resistance time to over 20 minutes. During the test, the swelling ratio was also impressive, reaching almost 50 times. And the thickness was 1 mm, which was also thin in the industrial field. Hence, the bi-layered design and optimized formulation are quite promising for practical application in high-end manufacturing.


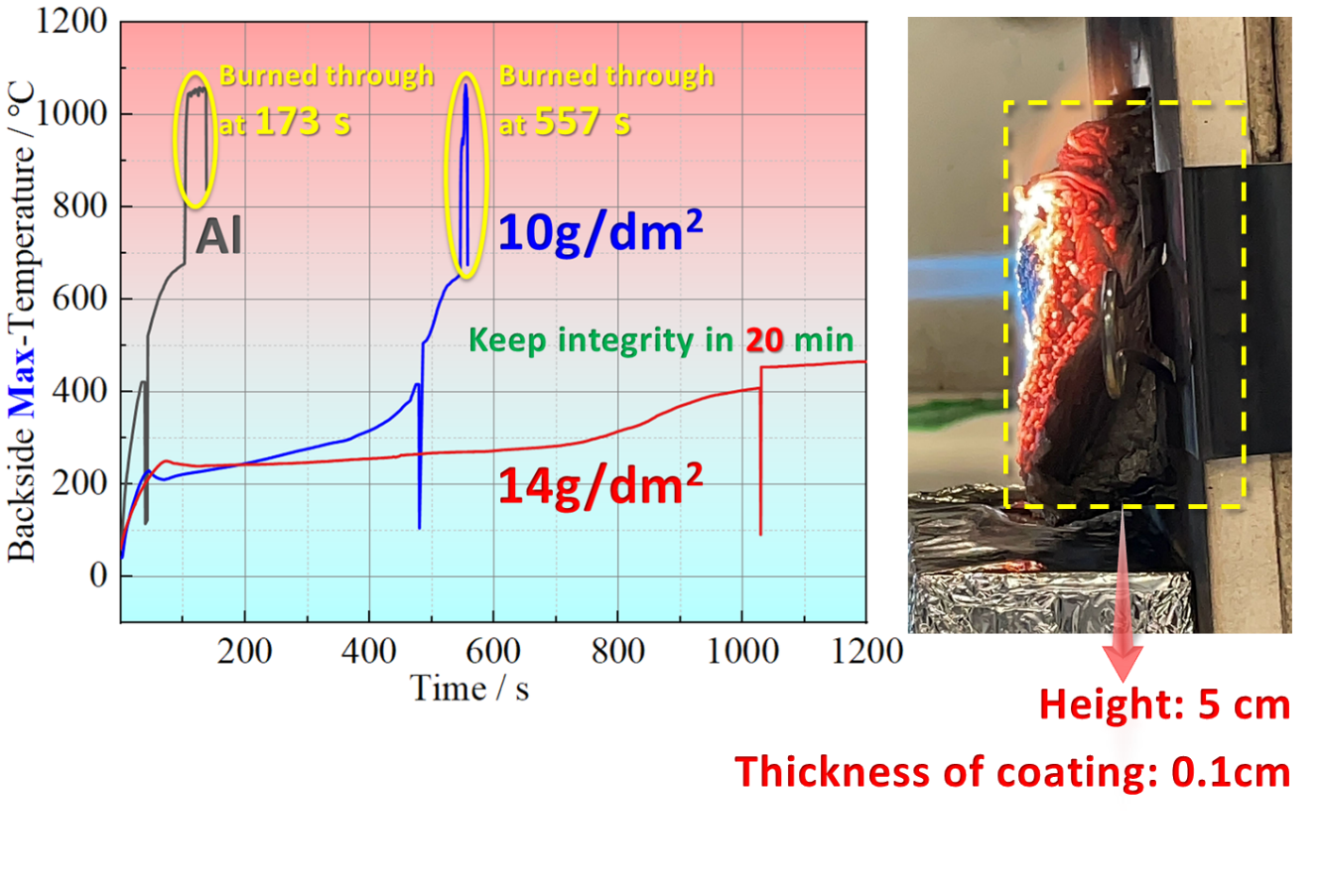


**Fig.** **S18** The burn-through results of EP based coating

The comparison shows that silicone rubber-based coatings are much more efficient than epoxy-based coatings, which may be explained by the difference in the thermal stability of the barrier layer. The silicone-based skeleton formed by the degradation of silicone rubber is more stable, allowing it to withstand high-temperature flame attacks. Epoxy degradation products, on the other hand, are mainly unsaturated carbon in structure and are prone to further degradation under sustained high-temperature flame damage, resulting in a coating that is more susceptible to burn-through. Nevertheless, the bi-layer strategy and formulation proposed in this work can still lead to a high efficiency of epoxy-based coatings.
